# Design of Hierarchical Porosity Via Manipulating Chemical and Microstructural Complexities in High‐Entropy Alloys for Efficient Water Electrolysis

**DOI:** 10.1002/advs.202105808

**Published:** 2022-02-24

**Authors:** Rui Li, Xiongjun Liu, Weihong Liu, Zhibin Li, K. C. Chan, Zhaoping Lu

**Affiliations:** ^1^ Northwestern Polytechnical University Xi'an 710072 P. R. China; ^2^ Beijing Advanced Innovation Center for Materials Genome Engineering State Key Laboratory for Advanced Metals and Materials University of Science and Technology Beijing Beijing 100083 P. R. China; ^3^ School of Materials Science and Engineering Harbin Institute of Technology Shenzhen Shenzhen 518055 P. R. China; ^4^ Advanced Manufacturing Technology Research Centre Department of Industrial and Systems Engineering The Hong Kong Polytechnic University Hong Kong P. R. China

**Keywords:** hierarchical pore, high‐entropy alloy, phase separation, spinodal decomposition, water electrolysis

## Abstract

Achieving a porous architecture with multiple‐length scales and utilizing the synergetic effects of multicomponent chemicals bring up new opportunities for further improving the electrocatalytic performance of nanocatalysts. Herein, the synthesis of a self‐supported hierarchical porous electrocatalyst based on a high‐entropy alloy (HEA) containing multiple transitional metals via physical metallurgy and dealloying strategies is reported. Microscale phase separation and nanoscale spinodal decomposition are modulated in a highly concentrated FeCoNiCu HEA, which makes it possible to obtain a porous structure with different length scales, i.e., relatively large porous channels formed by removing one separated phase and ultrafine mesopores obtained from leaching out one decomposition phase. The resultant hierarchical porous HEA exhibits superior water splitting performance, which takes full advantage of the enlarged surface area offered by the bi‐continuous mesoporous structure with the exceptional intrinsic reactivity originating from the synergetic electronic effects of the different components in alloying. Moreover, the microscale porous structure plays an important role in the significantly improved mass transportation, as well as the durability during electrocatalysis. This effective strategy that simultaneously utilizes the chemical and microstructural advantages of HEAs opens up a new avenue for developing HEA‐based, high‐performance porous electrocatalysts for various energy conversion/store applications.

## Graphical abstract

A self‐supported hierarchical porous high‐entropy alloy (HEA)‐based electrocatalyst is fabricated via physical metallurgy and dealloying strategies. Microscale phase separation and nanoscale spinodal decomposition are modulated in HEAs to obtain the porous structure with different length scales, which not only provides a large effective surface area but also accelerates charge and mass transport path for efficient water electrolysis.

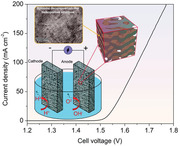

## 1 Introduction

High‐entropy alloys (HEAs) with a near equimolar fraction of multiple‐principal elements, although were initially developed as advanced structural materials, have attracted increasing attention in the field of catalysis, energy conversion and storage due to their strong alloying effect, high chemical disordering, and large lattice distortion.^[^ ^1^, ^2^, ^3^ ^]^ In particular, the designing of diverse HEAs is widely regarded as one of the most efficient strategies for developing novel catalysts for producing clean and renewable hydrogen energy, such as electrocatalytic water splitting.^[^ ^4^, ^5^, ^6^ ^]^ Previous studies demonstrated that transition metal‐based HEAs, such as NiFeMoCoCr, FeCoNiMn, and FeNiMnCrCu, exhibited promising electrocatalytic performance in hydrogen evolution reaction (HER) and/or oxygen evolution reaction (OER).^[^ ^7^, ^8^, ^9^ ^]^

The nanoporous or nanostructured HEAs produced by dealloying of alloys offer significantly enlarged specific surface areas.^[^ ^10^, ^11^ ^]^ Their potential applications for efficient electrocatalysis have been proposed recently.^[^ ^12^, ^13^, ^14^, ^15^, ^16^, ^17^, ^18^, ^19^, ^20^, ^21^ ^]^ However, the typical mesopore diameters and ligament size (usually <10 nm) could inherently impede the mass transferring and limit the reaction kinetics involving liquids and solids in the catalytic reactions.^[^ ^22^ ^]^ The mesoporous structure is also unfavorable to the gas release, increasing gas pressure inside the channels, which can eventually damage the pore structure.^[^ ^23^ ^]^ In this regard, a desirable porous HEA electrocatalyst requires a hierarchical porosity composed of both small and large pores to facilitate good electrochemical activity and fast mass transfer, respectively. The rapid ion diffusion will help to improve the overall reaction kinetics. As such, the development of porous architectures with multiple‐length scales yet containing various alloying metals using HEAs as precursors is of great importance for high‐efficient water splitting applications, both scientifically and technologically.

The multiple‐principal components (i.e., the compositional complexity) in HEAs offer a large composition variation for optimizing their phase structures. The high configuration entropy in HEAs also provides the possibility of manipulating hierarchical microstructures for obtaining multiporosity with desired length scales. Microscale microstructures due to phase separation were observed in several HEAs with simple lattices. Meanwhile, spinodal decomposition was also explored in various HEAs to create nanoscale dispersed phases. The formation of compositionally modulated microstructures was able to enhance the mechanical and magnetic properties of HEAs.^[^ ^24^, ^25^, ^26^ ^]^ Inspired by these studies, we attempted to investigate whether these physical metallurgy strategies in HEA are sufficient to manipulate their dealloyed porous architecture with enhanced electrocatalytic properties.

Herein, we report a reliable approach of developing a self‐supported dealloyed HEA electrocatalyst with pores at both micro and nanoscales for efficient hydrogen production. A quaternary FeCoNiCu HEA was selected as the dealloying precursor to produce the unique hierarchical porous architecture. The miscibility gap between Cu and the other components leads to the microscale phase separation and nanoscale spinodal decomposition structures. The dealloyed HEA electrocatalyst with a bicontinuous Cu‐rich dendritic‐like skeleton at the microscale with an internal secondary ultrafine mesoporosity exhibits outstanding HER performance. The low overpotential of 42.2 mV at a current density of 10 mA cm^−2^ and a low Tafel slope of 31.7 mV dec^−1^ from the dealloyed HEA electrocatalyst outperforms the commercial Pt/C catalysts as well as most other reported transition‐metal‐based compounds. The superior catalytic performance of the HEA‐based catalyst highlights the importance of the synergetic electronic effects of dissimilar metal atoms for reducing the HER energy barrier. More importantly, the size of the dealloyed hierarchical pore structures can be effectively controlled by manipulating the phase structure at different length scales in HEAs. Such a hierarchical porous structure provides not only a large accessible surface area but also a sufficient mass transport path for hydrogen evolution with superior structural stability. This work opens up a new paradigm for developing high‐performance electrocatalysts via properly designing and manipulating multiple length‐scale microstructures in HEAs.

## 2 Results and Discussion

The HEA precursor with a nominal composition of FeCoNiCu was fabricated by arc‐melting and Cu mold casting under an argon atmosphere. X‐ray diffraction (XRD) pattern in Figure S1 (Supporting Information) reveals an face‐centered cubic (FCC) lattice of the as‐cast FeCoNiCu HEA. The scanning electron microscopy (SEM) characterization (Figure S2a, Supporting Information ) exhibits a typical dendritic‐like microstructure of the as‐cast alloy, which is consistent with previous studies.^[^ ^27^ ^]^ Chemical compositions of the dendrites and interdendrites were estimated by energy‐dispersive X‐ray spectroscopy (EDS), as shown in Figures S2b,c (Supporting Information), it can be seen that the Cu element was enriched in the interdendritic regions due to the phase separation during solidification. The observed microscale phase separation is attributed to the positive mixing enthalpy of Cu with the other three elements, which is prone to segregate at grain boundaries.^[^ ^28^ ^]^ Moreover, a nanoscale spinodal decomposition feature was also observed in the inter‐dendritic region, evidenced by transmission electron microscopy (TEM) characterization (Figure S3, Supporting Information). The highly interconnected pattern of the spinodal structure exhibits an equiaxed shape with specific crystallographic orientations due to the small coherency‐strain energy associated with these directions.^[^ ^29^ ^]^ The Cu enriched and FeCoNi enriched regions with a similar dimension of ≈10 nm were formed due to the uphill diffusion of Cu atoms, accompanied by the compositional modulation. Their volume fractions can be clearly distinguished by the elemental mapping and EDS analyses using a high‐angle annular dark‐field scanning transmission electron microscope (HAADF‐STEM) (Figures S3c,d, Supporting Information). These results confirm the formation of multiple length‐scale phase structures in the as‐cast FeCoNiCu HEA.

**Figure 1:**
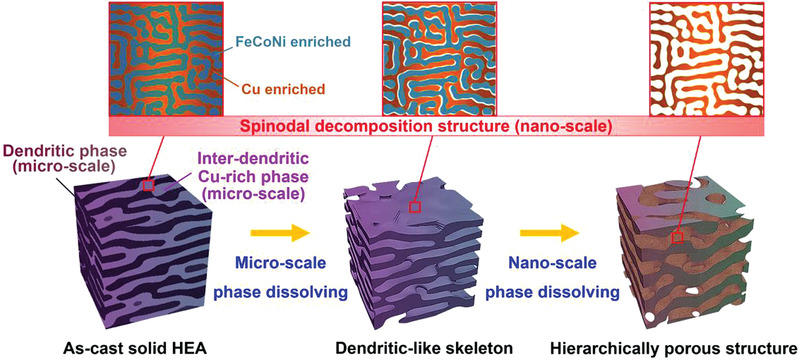
The schematic diagram for the architecture of the hierarchical porous structure via dealloying the FeCoNiCu HEA with microscale phase separation and nanoscale spinodal decomposition.

**Figure 2:**
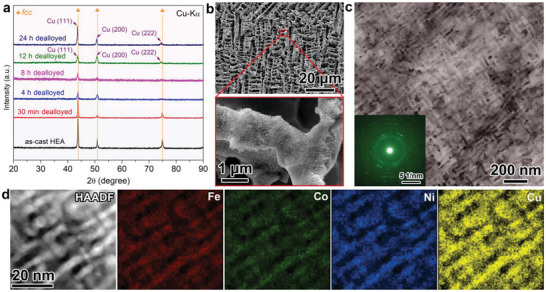
a) XRD patterns of the dealloyed FeCoNiCu HEA at different etching times. b) SEM characterization of the 8 h dealloyed FeCoNiCu HEA and the enlarged image of the ligaments with secondary mesoporosity. c) TEM images of the secondary spinodal decomposition‐like mesoporous structure. The inset shows the corresponding SAED pattern. d) HAADF‐STEM image and corresponding elemental mapping of the spinodal decomposition‐like mesoporous Cu enriched phase.

To obtain a porous HEA with hierarchical porosity, the as‐cast HEA sample was etched in an HNO_3_ solution (0.1 m) for selective dissolving. Surface morphology and structural evolution of the FeCoNiCu HEA during the dealloying process are shown in Figure S4 (Supporting Information). There are two separated phases at the microscale in the HEA with different corrosion resistances and chemical stability in the HNO_3_ etching. The Cu‐rich interdendritic phase is more inert than that of the dendritic phase due to the relatively high self‐corrosion potential of Cu.^[^ ^30^ ^]^ During the chemical dealloying process (Schematic diagram in **Figure** **1**), the dendritic phase with a lower Cu content was preferentially dissolved and the relatively stable Cu‐rich HEA inter‐dendritic phase was retained as the skeleton of the microscale porous structure. At the same time, a 3D secondary nanoporous structure with mesopore size was formed inside the dendritic‐like skeleton, which is resulted from the leaching of the FeCoNi enriched nanophase in the spinodal modulated structure. XRD patterns of the dealloyed HEAs with different etching durations are shown in **Figure** **2**a. It can be seen that the FCC peak intensity from the as‐cast HEA tends to decrease due to the continuous phase dissolution, accompanied by the peak broadening, indicating the formation of nanoscale grains by dealloying.^[^ ^13^, ^31^ ^]^ As the dealloying time was further increased (≥12 h), peaks originating from the crystalline Cu phase appeared as a result of the further dissolution of residual elements (i.e., Fe, Co, and Ni) in the Cu enriched nanophase. After 24 h dealloying, the FCC crystalline peaks from the as‐cast FeCoNiCu HEA were almost completely replaced by those from the Cu crystals, demonstrating the overetching of the secondary mesoporous structure, which can result in the subsequent collapse of the dendritic‐like skeleton at the micrometer scale. Figure 2b highlights the hierarchical porous structure formed after 8 h dealloying of HEA, which exhibits a 3D bi‐continuous microscale dendritic‐like morphology with an internal ultrafine secondary mesoporosity shown in the enlarged SEM image of the selected region, marked by the red square in Figure 2b. The size of the dealloyed porous channels, i.e., the so‐called skeletons is about 1.5 µm, corresponding to the dimension of the Cu‐rich inter‐dendritic phase in the as‐cast FeCoNiCu HEA. The average pore size of the secondary mesoporous structure inside the microscale skeleton is about 12.2 nm according to the Brunauer–Emmett–Teller (BET) measurement (Figure S5, Supporting Information). With the formation of the secondary nanoscale porosity, a greatly increased specific surface area of 14.6 m^2^ g^−1^ was obtained after 8 h dealloying, which is significantly higher than that of the as‐cast HEA sample (<0.05 m^2^ g^−1^). The microstructure of the dealloyed secondary mesoporous structure was also characterized by TEM, shown in Figure 2c. A typical mesoporous structure associated with spinodal decomposition was formed, in which the nanoscale ligaments of Cu enriched nanophase were interconnected in‐between the dissolved FeCoNi enriched nanophase (i.e., the observed mesopores, Figure S6, Supporting Information). Moreover, the ligament size is similar to the separation between the nanophases in the spinodal structure. The result demonstrates that the phase‐separated microstructure with spinodal modulated nanostructure can be effectively developed to form the hierarchical porous structure from the dealloyed HEA. The selected area electron diffraction (SAED) pattern (the inset in Figure 2c) and the high‐resolution TEM image (Figure S7, Supporting Information) confirm the polycrystalline nature of the dealloyed nanoscale ligaments. From the STEM elemental mappings in Figure 2d, the four‐element components with the relatively higher Cu content are homogeneously distributed in the secondary mesoporous structure. Moreover, the TEM‐EDS analysis (Figure S8, Supporting Information) and inductively coupled plasma‐optical emission spectrometry (ICP‐OES) measurement (Table S1, Supporting Information) demonstrate that the Cu content in the dealloyed hierarchical porous sample is close to 70 at%, which is consistent with the value in the initial nanoscale Cu enriched region resulting from the spinodal decomposition of the FeCoNiCu HEA. This result manifests that the mesoporosity is directly related to the nanoscale spinodal modulated structure in the as‐cast HEA. Moreover, the as‐cast FeCoNiCu HEA treated by rapid quenching (tube‐sealed and heated to 1200 °C) in water can suppress the uphill diffusion without the spinodal decomposition. The dealloying of this sample only produced a microscale porous structure without the secondary mesoporosity, shown in Figure S9 (Supporting Information), which confirms that modulating the spinodal decomposition structure in HEA is essential for the formation of the secondary mesoporous structure. XPS was also used to analyze the surface states of the as‐cast and dealloyed FeCoNiCu HEAs (Figures S10 and 11, Supporting Information, respectively), which demonstrate the coexistence of Fe, Co, Ni, and Cu, after the chemical dealloying. The presence of O in the dealloyed HEA sample is mainly attributed to surface oxidation of the reactive metallic elements. The evolved high‐valent states of the metallic elements correspond to the formation of metal oxides and metal hydroxides which can promote the OER,^[^ ^32^ ^]^ offering an opportunity for the application of water electrolyzer.

**Figure 3:**
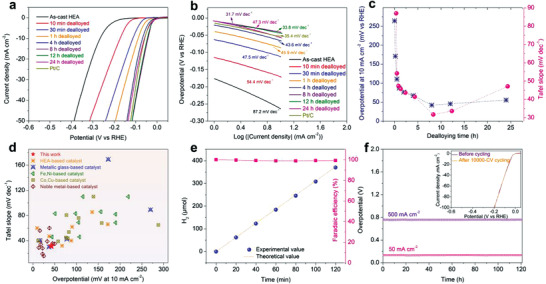
a) Polarization curves of the dealloyed FeCoNiCu HEA electrodes with different etching times in comparison with the Pt/C catalyst electrode in a 1.0 m KOH aqueous electrolyte. b) Tafel plots of the corresponding electrodes from the polarization curves. c) Variations of dealloying duration‐dependent HER performance (Left: overpotential at 10 mA cm^−2^; right: Tafel slope). d) Comparison of the HER performance of the optimal dealloyed HEA sample with various recently reported HER electrocatalysts in alkaline solutions. e) HER Faradaic efficiency of the 8 h dealloyed HEA electrode in 1.0 m KOH electrolyte at a current density of 10 mA cm^−2^. f) Long‐term stability measurement of the dealloyed HEA electrode at the current densities of 50 and 500 mA cm^−2^, respectively, for more than 120 h. The inset shows the HER polarization curves of the dealloyed HEA electrode before and after 10 000‐CV cycling.

To evaluate the electrocatalytic performance of the prepared hierarchical porous HEA‐based catalysts, all dealloyed HEA samples with different etching durations were directly used as the working electrodes in a 1.0 m KOH electrolyte. **Figure** **3**a compares the typical HER polarization curves of the dealloyed electrodes in comparison with the as‐cast FeCoNiCu HEA and commercial Pt/C catalysts. The dealloyed HEA electrodes exhibited significantly enhanced HER catalytic activities due to the porous structure. The 8 h dealloyed HEA exhibited the lowest overpotential of 42.2 mV at the current density of 10 mA cm^−2^, which is even lower than that of the commercial Pt/C catalyst by 13 mV. The corresponding Tafel slopes are shown in Figure 3b, where the superior electrocatalytic HER activity of the dealloyed HEAs is manifested by the low Tafel slope, in association with the Volmer‐Tafel mechanism. Figure 3c demonstrates the relationship between the etching time and the electrocatalytic performance. The HER activity improves as the dealloying time increases from 0 to 4 h. This is attributed to the creation of the Cu‐rich dendritic‐like structure at the initial stage of selective dissolution (Figures S4a–f, Supporting Information), which provides more active sites for hydrogen evolution. The HER activity of the dealloyed sample was further improved when the 3D bi‐continuous hierarchical porous structure was formed (i.e., the 8 h dealloyed HEA, Figure S4g, Supporting Information). In this state, the sample offered a much larger contact area with abundant active sites for the catalytic reaction following the formation of the secondary spinodal decomposition‐like mesoporous structure with a high density of mesopores. The dendritic‐like skeleton with macropores also helped to maintain fast mass transportation and ion diffusion during the electrocatalysis, resulting in the further optimized HER kinetics. Moreover, the lower Tafel slope of the hierarchical porous HEA than that of the microscale porous sample without the secondary mesoporosity (Figure S12, Supporting Information) reveals that the formation of the secondary mesoporous structure cannot only offer an enlarged internal surface area for exposing active sites to accelerate the hydrogen evolution, but also generate more efficient active sites due to the composition change of remnant ligaments caused by the leaching out of one nanoscale decomposition phase in the HEA, further improving the intrinsic HER activity. This result is also confirmed by the true steady‐state Tafel plots (Figure S13, Supporting Information) calculated according to the galvanostatic response to avoid the scan rate effect.^[^ ^33^ ^]^ However, further increasing the dealloying duration (>12 h) has caused the deterioration of the electrocatalytic activity. At such a long dealloying process, the residual Fe, Co, and Ni elements were selectively removed from the Cu enriched nanophase, leading to a thinner ligament formed with only Cu, while some dendritic‐like porous structure collapsed, demonstrated by the XRD in Figure 2a and SEM in Figure S4i (Supporting Information). The absence of synergetic chemical effects from multicomponent nature and the decrease of the effective contact areas are detrimental to the electrocatalytic performance. The reaction kinetics of the dealloyed samples was further studied by the electrochemical impedance spectroscopy (EIS) and electrochemically active surface area (ECSA) measurements. Figure S14 (Supporting Information) presents typical Nyquist plots obtained at the overpotential of 100 mV with the high‐frequency regions shown in Figure S15a (Supporting Information). The EIS data were fitted to an equivalent resistor–capacitor circuit model including the resistance of electrolyte solution (*R*_s_), a charge transfer resistance (*R*_ct_), and a double‐layer capacitance (*C*). The EIS results revealed that the dealloyed HEA with a 3D hierarchical porosity offered the lowest *R*_ct_ (Figure S15b, Supporting Information), implying that the multicomponent nature and the bi‐continuous hierarchical porous structure offered the optimized electronic structure and diffusion kinetics resulting in the best HER kinetics. The ECSA measured by the double‐layer capacitance method (Figures S16 and 17, Supporting Information) also confirmed the presence of enlarged contact surface area with more active sites for the enhanced hydrogen evolution HER performances from the dealloyed HEAs with hierarchical porous structure. In addition, the HER activity of the dealloyed FeCoNiCu HEA without hierarchical porosity is much better than that of the dealloyed porous binary NiCu and ternary FeNiCu alloy catalysts (Figure S18, Supporting Information), further demonstrating the substantial contribution of the synergetic electronic effects from multiple metal constituents (i.e., the high‐entropy nature) to the intrinsic catalytic reactivity. When compared with recently reported catalytic HER results in alkaline electrolytes, including HEAs or HEAs with a simple nanoporous structure, metallic glasses, transition metals, and noble metal‐based materials, it is encouraging to see that our 3D hierarchical porous HEA‐based catalyst offered a high HER performance, even better than some noble metal‐based electrocatalysts (Figure 3d and Table S2, Supporting Information). Considering the economic benefit of the non‐noble metal element and the simplicity of the preparation method, the newly developed HEA catalyst can serve as a promising electrocatalyst candidate for practical water splitting applications.

In addition to the electrocatalytic activity, the catalyst durability and stability are also important for practical water splitting applications. In this work, the reliability of the 8 h dealloyed HEA was assessed. Figure 3e shows the Faradaic efficiency variation of the porous catalyst towards HER at different reaction durations. The Faradaic efficiency is determined by comparing the experimental H_2_ production with the theoretically calculated value from the current (Table S3, Supporting Information). The near 100% Faradaic efficiencies indicate that no side reaction took place during the electrolysis, confirming the high efficiency and durability. The stability was also tested using chronopotentiometry measurements at the current densities of 50 and 500 mA cm^−2^ for 120 h, respectively (Figure 3f), the overpotentials showed no observable increase due to the stable dendritic‐like porous morphology and the good corrosion resistance of the HEA in the alkaline electrolyte, as proved by SEM characterization (Figure S19, Supporting Information). The high‐resolution XPS spectra (Figure S20, Supporting Information) also reveal that the surface elements keep the almost same chemical states as the initial ones after the durability test, demonstrating the remarkable electrochemical stability. An accelerated CV cycling test shows a negligible shift of the polarization curves after 10,000 cycles (the inset in Figure 3f), which also verifies the stable catalytic HER performance.

**Figure 4:**
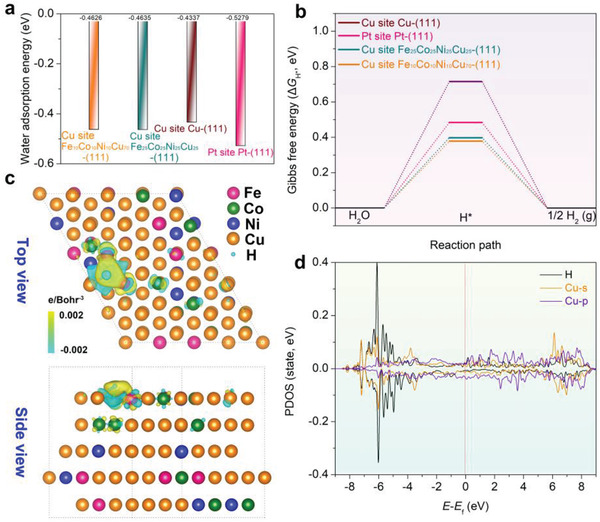
a) Comparison of the adsorption energies of H_2_O molecule on the optimal sites of the Fe_10_Co_10_Ni_10_Cu_70_‐(111), Fe_25_Co_25_Ni_25_Cu_25_‐(111), pure Cu‐(111), and Pt‐(111) surfaces. b) Gibbs free energies for hydrogen adsorption at different optimal active sites of the catalyst surfaces. c) Computed differential charge densities for hydrogen adsorption on the optimal Cu site of the Fe_10_Co_10_Ni_10_Cu_70_‐(111) surface. Yellow and blue bubbles represent the positive and negative charges with an iso‐value of 0.002 e Å^−3^, respectively. d) PDOS of the Fe_10_Co_10_Ni_10_Cu_70_‐(111) surface after the H* adsorption on the optimal Cu site. The red dotted lines at the energy of zero indicate the Fermi level.

The ultrafine nanoscale secondary porosity and the microscale skeleton of the dealloyed hierarchical porous structure offer a high specific surface area with fast charge transfer and mass transportation for heterogeneous electrocatalysis. The intrinsic reactivity of the dealloyed HEA towards HER is also enhanced due to the synergetic chemical effects. Density‐functional theory (DFT) calculations were conducted to confirm such mechanisms for the enhanced intrinsic HER activity. It is noted that surface oxidation of the alloy surface is unavoidable, herein, we focused on the synergistic chemical effect of metallic Fe, Co, Ni, and Cu towards the hydrogen evolution without consideration of surface oxidation which mainly affects the OER process based on previous studies.^[^ ^20^, ^32^ ^]^ According to the TEM EDS and ICP‐OES analyses (Figure S8 and Table S1, Supporting Information), a solid solution with a chemical composition of Fe_10_Co_10_Ni_10_Cu_70_ was selected as the model for dealloyed HEA with exposed (111) facet. The kinetic energy barriers of the HER process were calculated and compared with the unetched Fe_25_Co_25_Ni_25_Cu_25_‐(111), pure Cu‐(111), and pure Pt‐(111) surfaces. The adsorption energy for H_2_O on the optimal Cu active site of the Fe_10_Co_10_Ni_10_Cu_70_‐(111) surface was also calculated (Figure S21, Supporting Information). In comparison with various active sites in different model materials (Figure S22 and Table S4, Supporting Information), the optimal Cu sites on the Fe_10_Co_10_Ni_10_Cu_70_‐(111) and Fe_25_Co_25_Ni_25_Cu_25_‐(111) surfaces present comparable H_2_O adsorption energies (−0.463 and −0.464 eV, respectively), which are more negative than that of the pure Cu‐(111) (−0.434 eV) (**Figure** **4**a). This suggests that the presence of other metal elements improves the adsorption of H_2_O molecules and the Volmer process of HER in the alkaline electrolyte (i.e., the reaction of * + H_2_O + e^‐^ → H* + OH^‐^, where * represents active sites) due to the synergetic chemical effect of multiple components. Additionally, the hydrogen adsorption Gibbs free energy (Δ*G*_H*_), representing the theoretical activity for HER, was also calculated (Figure 4b). The lower Δ*G*_H*_ value is associated with a lower adsorption/desorption energy barrier of H*. The lowest Δ*G*_H*_ value of 0.378 eV was found at the optimal Cu site surrounded by other non‐Cu metals on the Fe_10_Co_10_Ni_10_Cu_70_‐(111) surface in an alkaline electrolyte (corrected by the pH value) (Figure S23, Supporting Information). A slightly higherΔ*G*_H*_ value of 0.398 eV was found on the Fe_25_Co_25_Ni_25_Cu_25_‐(111). These values are much lower than those of pure Cu‐(111) (0.716 eV) and even the Pt‐(111) (0.484 eV) (Figure S24 and Table S5, Supporting Information). Hence the synergetic chemical effect from the mixing of multiple metal elements on the alloy surface is responsible for reducing the energy barrier of the adsorption/desorption of H*, resulting in the improved HER kinetics. The charge density difference before and after the H* adsorption (Figure 4c) also reveals a stronger charge redistribution around the Cu adsorption site on the Fe_10_Co_10_Ni_10_Cu_70_‐(111) in comparison with the pure Cu‐(111) (Figure S25, Supporting Information). This is associated with the chemical complexity of HEAs, which promotes the charge transfer from a Cu active site to the H*, resulting in superior HER kinetics. Figure 4d shows the calculated partial density of states (PDOS) of the Fe_10_Co_10_Ni_10_Cu_70_‐(111) surface after the H* adsorption. The PDOS of the Cu *s*‐orbitals is aligned with that of the H *s*‐orbitals, offering a strong electronic interaction between the Cu and the H atoms with a high density of PDOS overlap in comparison with the pure Cu‐(111), which is important for the reduction of H* on the Cu sites (Figure S26, Supporting Information). Thus, the cooperation of Fe, Co, and Ni atoms improves the electronic structure of Cu atoms for interaction with H*, resulting in a fast hydrogen evolution process. A similar result was also achieved for Fe_25_Co_25_Ni_25_Cu_25_‐(111) (Figure S27, Supporting Information). The above results demonstrate that the chemical complexity of the HEA alloy has positive effects on the catalytic HER reactivity. Therefore, the synergistic chemical effect in the dealloyed porous structure was originated from the appropriate surface state with the reduced energy barriers of HER.

**Figure 5:**
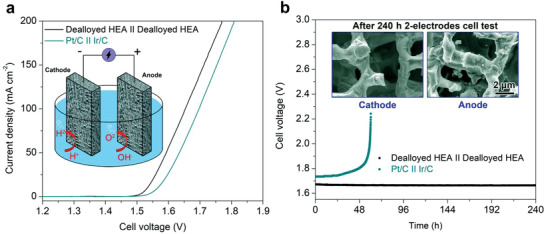
a) Polarization curves of electrocatalytic water electrolysis using (dealloyed HEA)||(dealloyed HEA) electrodes (black) and Pt/C||Ir/C electrodes (olive), respectively. The inset shows the schematic illustration of the water electrolyzer, constructed with the dealloyed HEA electrodes as both cathode and anode. b) Durability tests of the water electrolysis with the (dealloyed HEA)||(dealloyed HEA) electrodes (black) and the Pt/C||Ir/C electrodes (olive) at the fixed current density of 100 mA cm^−2^. The insets show the SEM images of the dealloyed HEA cathode (left) and anode (right) after the 240 h durability test, respectively.

Besides the excellent intrinsic HER activity, the hierarchical porous HEA catalyst also exhibited impressive OER performance in the alkaline electrolyte (Figure S28, Supporting Information), which may be benefitted from the formation of the surface oxides and hydroxides during the dealloying process (Figure S10, Supporting Information). Hence, we examined the overall water splitting performance and stability of the hierarchical porous HEA‐based catalyst to evaluate its potential for practical water electrolysis. Efficient generation of hydrogen (cathode) and oxygen (anode) gases using the porous HEA‐based catalysts as both cathode and anode was confirmed, illustrated in Movie S1 (Supporting Information). **Figure** **5**a presents the polarization curves of the two‐electrode electrolyzer constructed with the dealloyed porous HEA catalysts in 1.0 m KOH electrolyte (the inset shows the schematic illustration of the electrolyzer powered by electrical energy) in comparison with the cell using commercial Pt/C for the cathode and the Ir/C for the anode. At the current density of 10 mA cm^−2^, the HEA‐based catalyst offered the lowest water electrolysis potential of 1.53 V. This is lower than the electrodes made from the commercial catalysts (Pt/C||Ir/C, 1.56 V), and is also lower than many reported transition metal‐based catalysts such as the FeCoNi nanosheets/CC||FeCoNi nanosheets/CC (1.55 V),^[^ ^34^ ^]^ NiCo_2_O_4_||NiCo_2_O_4_ (1.61 V),^[^ ^35^ ^]^ and Cu_3_N||Cu_3_N (1.60 V).^[^ ^36^ ^]^

The long‐term water splitting performance was also tested at a higher current density of 100 mA cm^−2^, shown in Figure 5b. The cell potential was stable at about 1.67 V without any appreciable change of the potential during the continuous electrolysis for longer than 240 h. Moreover, the anode and cathode electrodes still maintained their initial microstructures after the long‐term measurement (the inset in Figure 5b). For the Pt/C||Ir/C cell, however, a rapid increase in the potential was observed after only about 59 h under the same test condition, which is associated with the decreased water electrolysis efficiency. The result verifies the excellent operational durability of the dealloyed porous HEA as the electrocatalyst for water electrolysis. XPS analysis was also performed to investigate the chemical state changes of the surface metal elements on the OER electrode after the 240 h continuous electrolysis. As shown in Figure S29 (Supporting Information), although the oxidation of the surface Fe, Co, Ni, and Cu elements has occurred during the OER process, the integrity of the electrode was well preserved. From the O 1*s* signal, the intensity ratio between the metal‐OH/OOH and metal–O was increased after the electrolysis in comparison with the initial one (Figure S11, Supporting Information). Therefore, the OH/OOH‐rich active intermediates were formed on the dealloyed HEA surface, which contributes positively to the OER activity.^[^ ^37^, ^38^ ^]^ In addition, the energy efficiency at 100 mA cm^−2^ of water electrolysis using the (dealloyed HEA)||(dealloyed HEA) configuration was 83.4% according to the method in the literature,^[^ ^39^, ^40^ ^]^ which is much higher than that of the typical noble‐metal‐based water electrolysis (≈70%). Even considering the industrial operating stability as an important metric, the assembled cell can still keep an average voltage of 1.95 V at a large current density of 500 mA cm^−2^ over 240 h (Figure S30, Supporting Information). Although slight performance degradation was observed with the operating time further prolongs, the electrode structure is well preserved without significant physical damages or detachment. This observation demonstrates that the dealloyed HEA catalyst with a hierarchical porous structure has great potential for practical water splitting.

## 3 Conclusions

In summary, we have developed a hierarchical porous structure by dealloying HEA, which was used as the high‐efficiency water splitting electrocatalyst. The microstructure of the dealloyed HEA shows the coexistence of a microscale dendritic‐like skeleton and an internal secondary spinodal decomposition‐like mesoporous structure formed with multiple metal elements. The dealloyed Cu enriched nanophase shows the advantage of synergetic chemical effect, offering the appropriate electronic structure for exceptionally intrinsic electrocatalytic reactivity for HER. The hierarchical porous morphology with multiple‐length scales not only provides a large effective surface area but also accelerates charge and mass transport path for fast hydrogen evolution. As a result, the self‐supported HEA‐derived porous electrode exhibits superior HER performance in alkaline electrolytes, with a low overpotential of 42.2 mV to achieve a current density of 10 mA cm^−2^, in addition to a low Tafel slope of 31.7 mV dec^−1^. The sample also showed excellent HER stability under a high current density of 500 mA cm^−2^. Moreover, the HEA‐derived porous electrode could also be used as the anode material for efficient OER. A cell using the dealloyed HEA as both cathode and anode was constructed which showed good reliability for long‐term water splitting performance. This work explored the potential of utilizing the chemical and structural complexity in HEAs via physical‐metallurgy engineering for developing promising hierarchical porous electrocatalysts for wide energy conversion applications.

## 4 Experimental Section

### Material Fabrication

The HEA with a nominal composition of Fe_25_Co_25_Ni_25_Cu_25_ (at%) was prepared by the arc‐melting and copper mold casting method. The alloy ingots were prepared by arc‐melting a mixture of high purity (99.99 wt%) elements of Fe, Co, Ni, and Cu under Ti‐gettered argon atmosphere and remelted at least four times to ensure chemical homogeneity. Then, the melted master ingots were drop‐casted into a copper mold with a dimension of 5 mm × 12 mm × 600 mm. The resultant as‐cast HEA plates were then cut and polished into 10 mm × 5 mm × 30 µm segments for the subsequent dealloying, characterization, and electrochemical testing. For the chemical etching, the prepared HEA samples were treated in an HNO_3_ aqueous solution (0.1 m) at different time durations for selective dissolution. The dealloyed HEA samples were repeatedly ultrasonicated in deionized water and dehydrated alcohol to remove residual chemical substances and then preserved in a vacuum oven to prevent oxidation until further characterization and electrochemical testing.

### Structural Characterization

The phase structure of samples and the structural evolution during the dealloying process were determined by X‐ray diffraction (XRD, Rigaku D‐max 2500/PC, Cu‐K*α*). Microstructural analyses of the as‐cast HEA and the dealloyed HEA samples were conducted using a scanning electron microscope (SEM, FEI Quanta450FEG) equipped with energy‐dispersive X‐ray spectroscopy (EDS) and a transmission electron microscope (TEM, JEOL‐2100F) coupled with selected area electron diffraction (SAED). The TEM specimen of the as‐cast HEA was prepared by the focused‐ion beam method. The dealloyed sample was dispersed by sonication in dehydrated alcohol and the resultant suspensions were put onto holey carbon‐supported molybdenum grids for the TEM study. X‐ray photoelectron spectroscopy (XPS, Thermo Escalab 250Xi) with an Al K*α* anode at an energy level of 150 W was employed to probe the chemical state and the binding energies of the elements in the samples. All binding energies were calibrated at the C 1*s* position (284.4 eV) from the contaminant carbon in the vacuum chamber of the instrument. The specific surface areas of the dealloyed HEA samples were measured by an automated surface area and pore size analyzer (Quadrasorb SI). ICP‐OES (Agilent Technologies 5110) was used to determine the composition of the HEA samples before and after dealloying.

### Electrochemical Measurement

All electrochemical measurements were performed on an electrochemical workstation (CHI 660D) using a three‐electrode cell equipped with a graphite rod as the counter electrode and an Ag/AgCl electrode as the reference electrode. The bare HEA and the dealloyed HEA samples with a fixed size of 10 mm × 5 mm × 30 µm were directly used as the working electrodes. The commercial Pt/C catalyst ink was used for comparison by dispersing Pt/C (20 mg, 20 wt%, Sigma‐Aldrich) into a mixture solution containing water/ethanol (490 µL, 1:1) and Nafion (10 µL, 5 wt%, Sigma‐Aldrich). The solution was mixed by ultrasonication for 1 h before drop‐cast of 2 µL onto a 3 mm glassy carbon electrode (GCE). The sample was dried at room temperature with the typical catalyst loading of about 1.13 mg cm^−2^. The electrocatalytic measurements were conducted in an O_2_ saturated KOH aqueous solution (1.0 m). For convenience, all potentials were converted to the RHE using the Nernst equation: *E*_RHE_ = *E*_Ag/AgCl_ + 0.0591   ×   pH + *E* ^0^_Ag/agCl_ (PH = 13.89,^[^ ^41^ ^]^ *E* ^0^_Ag/AgCl_= 0.197 V versus normal hydrogen electrode, *T* = 25 °C). Electrochemical impedance spectroscopy (EIS) spectra were obtained with a frequency ranging from 10^6^ Hz to 0.01 Hz with an amplitude of 10 mV. All linear scan voltammetry curves were conducted at a scan rate of 2 mV s^−1^ and corrected for *iR* losses. The *iR* correction of polarization curves was performed using the solution resistance (*R*_s_) estimated from EIS measurements. The potential was based on *iR* correction using the equation: *E*(*iR* − corrected) = *E* − *iR*, where *i* is the current and *R* is the uncompensated electrolyte Ohmic resistance measured by EIS. Electrochemical double‐layer capacitance was measured to determine the electrochemically active surface areas (ECSAs) of the catalysts at non‐faradaic overpotentials, according to our previously reported method.^[^ ^42^ ^]^ Faradaic efficiency was calculated by the ratio between the measured amount of H_2_ generated by electrolysis and the calculated amount of H_2_ based on the current density.^[^ ^43^ ^]^ The HER durability was measured by repeating the potential scan from −0.3 to 0 V versus reversible hydrogen electrode (RHE) at a sweep rate of 100 mV s^−1^ with 10 000‐CV cycles. Chronoamperometric characterization was also performed at current densities of 50 and 500 mA cm^−2^ for 120 h. For the water electrolysis, a two‐electrode cell was used. Both anode and cathode used the dealloyed HEA samples to assess the OER and HER performance in a 1.0 m KOH electrolyte. The chronopotentiometry tests under the current density of 100 mA cm^−2^ were carried out to evaluate the durability of the electrode material. For further electrocatalytic activity comparison, binary NiCu and ternary FeNiCu alloy were also prepared and dealloyed under the same conditions and used as working electrodes for electrochemical tests.

### Theoretical Calculation

First‐principles calculations were carried out based on density‐functional theory (DFT) as implemented in the Vienna ab initio simulation package (VASP).^[^ ^44^, ^45^ ^]^ The ion‐electron interactions were described using the projector‐augmented‐wave (PAW) approach. For the exchange‐correlation functional, the generalized gradient approximation (GGA) of the Perdew–Burke–Ernzerhof (PBE) approach was employed. The cutoff energy was set to be 400 eV. The semi‐empirical DFT‐D3 approach developed by Grimme was employed to evaluate the Van der Waals interactions. The convergence criteria of energy and force were set to 10^−6^ eV atom^−1^ and 0.02 eV Å^−1^, respectively. The 12 × 12 × 12 and 11 × 11 × 11 gamma centered k‐points with the Monkhorst–Pack scheme were used for the pure FCC Cu and Pt, respectively. The p(4 × 5) models of Cu (111) and Pt (111) slabs with 5 atomic layers and 100 atoms were used to simulate the pure metal surface. The Fe_10_Co_10_Ni_10_Cu_70_ and Fe_25_Co_25_Ni_25_Cu_25_ solid solution surfaces were constructed by randomly replacing Cu atoms in the Cu (111) slab with Co, Fe, and Ni atoms. For all surface slabs, a vacuum layer of 15 Å was added in the *Z* direction to avoid the interaction between periodic slabs. A 3 × 2 × 1 gamma centered k‐point with the Monkhorst–Pack scheme was used for all surface slabs. During the surface slab calculations, the bottom three layers of atoms were fixed.

In this work, the HER process in the alkaline electrolyte was considered as follows: 1) * + H_2_O + e^−^ → H* + OH^−^and 2) H* + H_2_O + e^−^ → H_2_(g) + OH^−^, where the asterisk (*) represents the surface substrate active site. The water adsorption energy (*E*_ads_) is defined as: $E_{H2O\_\mathrm{ads}\_\mathrm{surface}}$, *E*_surface_, and *E*_H2O_ are the energies of H_2_O adsorbed on the surface, the clean surface, and the H_2_O in a 10 × 11 × 12 box, respectively. Here, a more negative *E*_ads_ suggests easier adsorption of water molecules. The free energies of the HER steps were calculated by the equation: Δ*G*_H*_ = Δ*E*_H*_ +  Δ*E*_ZPE_ − *T*Δ*S*, where Δ*E*_H*_ is the DFT electronic energy difference of each step, Δ*E*_ZPE_ and Δ*S* are the correction of zero‐point energy and the change of entropy, respectively, obtained by vibration analysis. *T* is the temperature (*T* = 298.15 K).

## Conflict of Interest

The authors declare no conflict of interest.

## Supporting information

Supporting Information

Supplemental Movie 1

## Data Availability

The data that support the findings of this study are available from the corresponding author upon reasonable request.
