# Supplementary material for: Design of Hierarchical Porosity Via Manipulating Chemical and Microstructural Complexities in High‐Entropy Alloys for Efficient Water Electrolysis
Source: Adv Sci (Weinh). 2022 Feb 24;9(12):2105808. doi: 10.1002/advs.202105808 (PMC9036019; doi:10.1002/advs.202105808)
Supplement: Supplementary file 1 — Supporting Information [file ADVS-9-2105808-s002.pdf]

Supporting Information

Design of Hierarchical Porosity via Manipulating Chemical and Microstructural Complexities in High-Entropy Alloys for Efficient Water Electrolysis

Rui Li, Xiongjun Liu, Weihong Liu, Zhibin Li, K. C. Chan*, and Zhaoping Lu**

Dr. R. Li
Northwestern Polytechnical University
Xi'an, 710072, P. R. China

Prof. X. J. Liu, Z. B. Li, Prof. Z. P. Lu
Beijing Advanced Innovation Center for Materials Genome Engineering, State Key Laboratory for Advanced Metals and Materials
University of Science and Technology Beijing
Beijing 100083, P. R. China
E-mail: xjliu@ustb.edu.cn; luzp@ustb.edu.cn

Dr. W. H. Liu
School of Materials Science and Engineering
Harbin Institute of Technology
Shenzhen, P. R. China

Prof. K. C. Chan
Advanced Manufacturing Technology Research Centre
Department of Industrial and Systems Engineering
The Hong Kong Polytechnic University, Hong Kong
E-mail: kc.chan@polyu.edu.hk

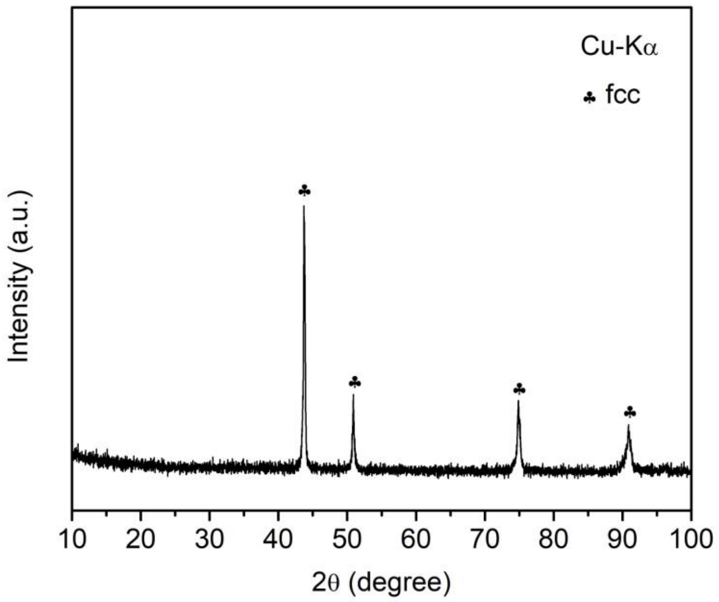

Figure S1. XRD pattern of the as-cast FeCoNiCu HEA.

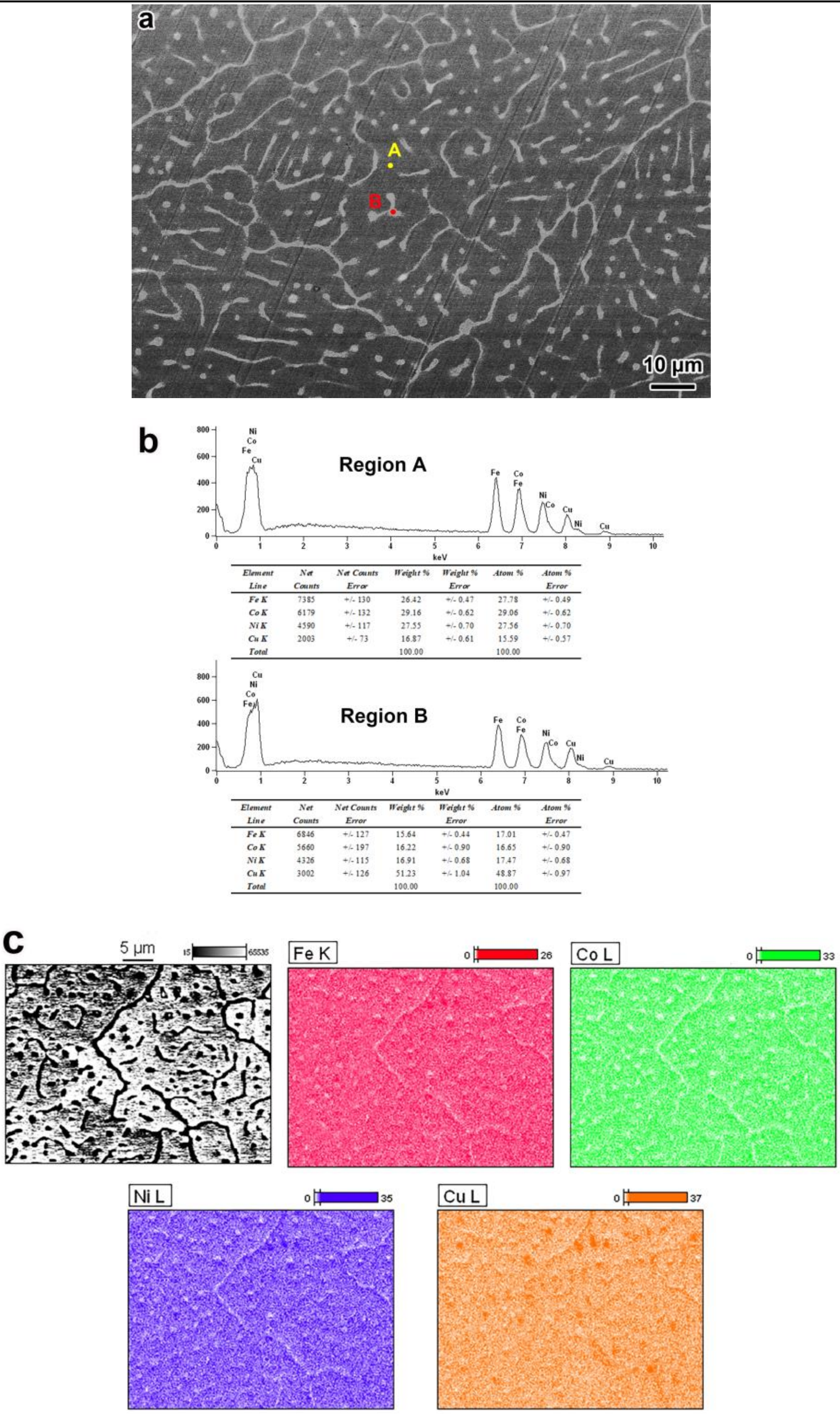

Figure S2. (a) BSE image of the as-cast FeCoNiCu HEA. (b) EDS analysis of different regions marked in the SEM image of the as-cast FeCoNiCu HEA. Region A: dendritic phase; Region B: inter-dendritic Cu-rich phase. (c) EDS mapping scanning of the as-cast FeCoNiCu HEA surface.

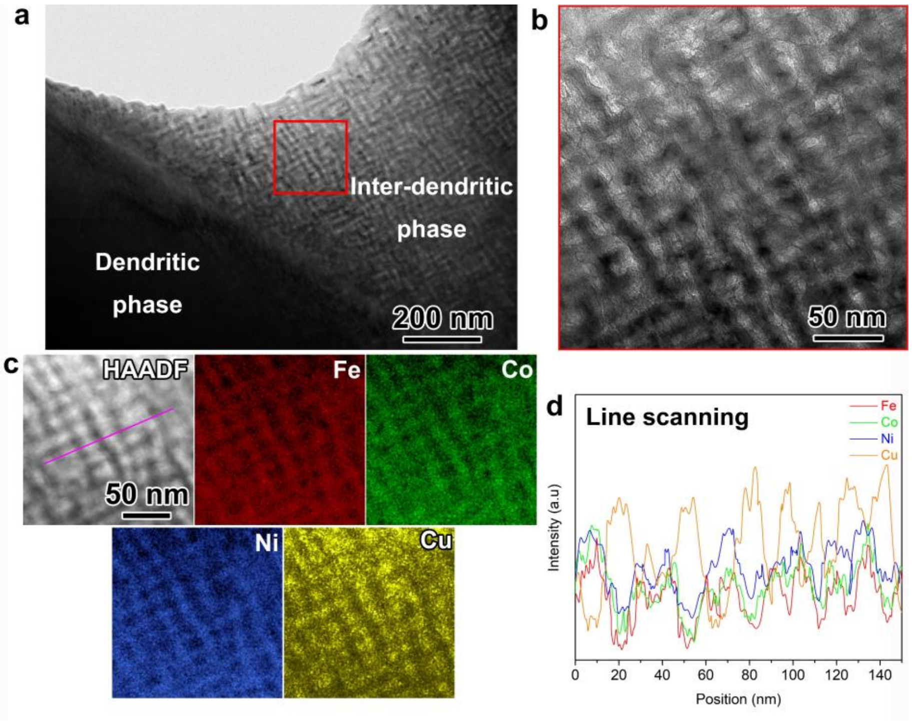

Figure S3. TEM characterization of the as-cast $Fe_{25}Co_{25}Ni_{25}Cu_{25}$ HEA precursor: (a) TEM image of the interface of the dendritic and inter-dendritic regions, the spinodal decomposition structure can be distinguished in the Cu-rich inter-dendritic phase. (b) Enlarged TEM image of the spinodal decomposition structure marked in (a) by a red box. (c) HAADF-STEM image and corresponding elemental mapping of the spinodal decomposition structure of the as-cast FeCoNiCu HEA. (d) Line scanning analysis of the nano-scale spinodal decomposition structure along the pink line marked in (c).

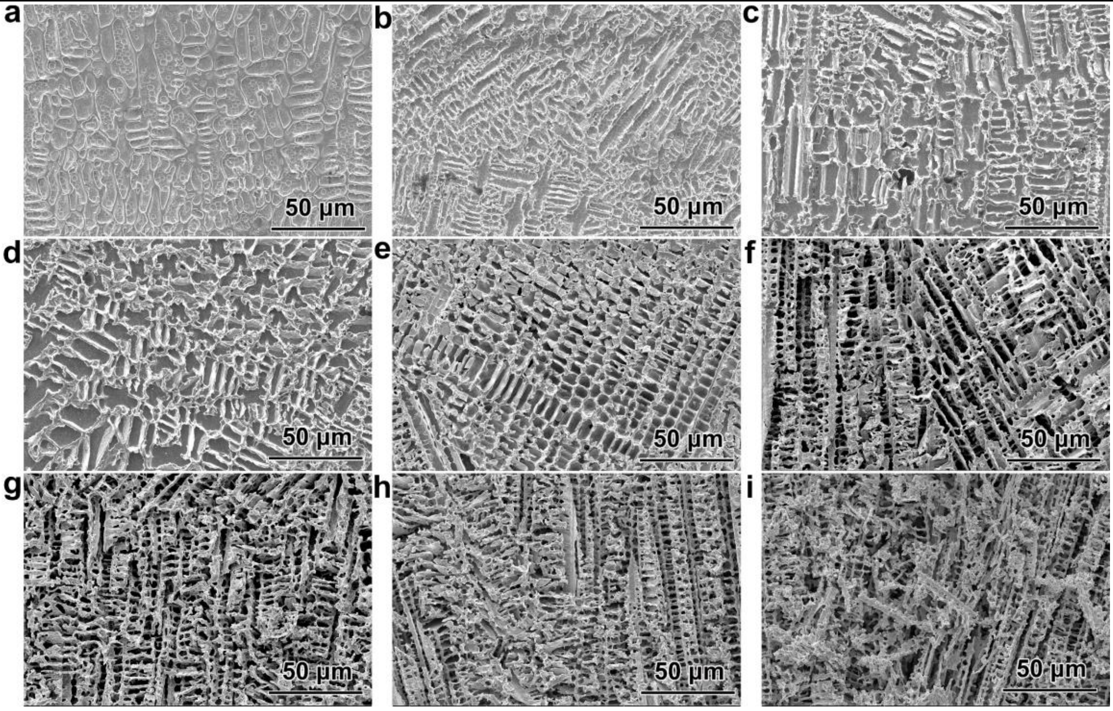

Figure S4. Surface structural evolution of the dealloyed FeCoNiCu HEA with different etching durations in 0.1 M HNO_3 aqueous solution: (a) 10 min, (b) 20 min, (c) 30 min, (d) 1 h, (e) 2 h, (f) 4 h, (g) 8 h, (h) 12 h, and (i) 24 h.

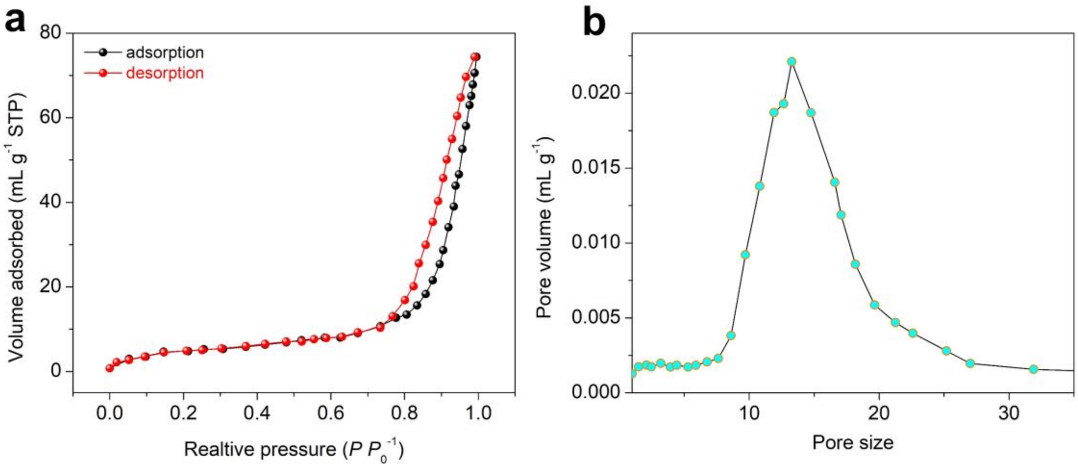

Figure S5. (a) N_2 adsorption and desorption isotherm curves of the 8 h dealloyed HEA. (b) The pore size distribution of the 8 h dealloyed HEA sample.

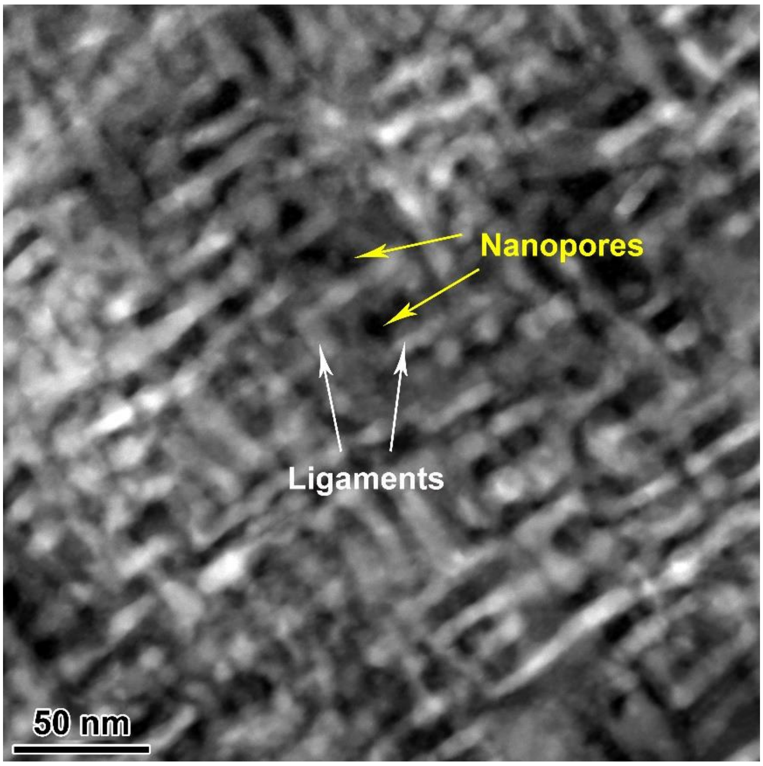

Figure S6. Enlarged TEM morphology of the pores and ligaments of the dealloyed secondary mesoporous structure.

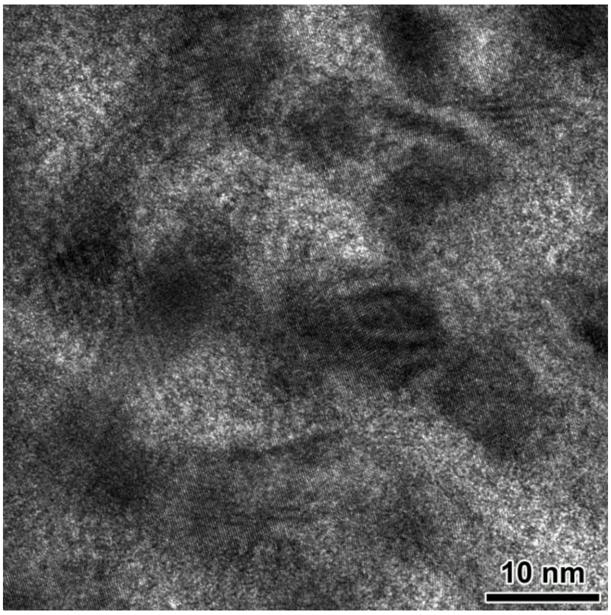

Figure S7. High-resolution TEM image of the secondary mesoporous structure of the 8 h dealloyed FeCoNiCu HEA.

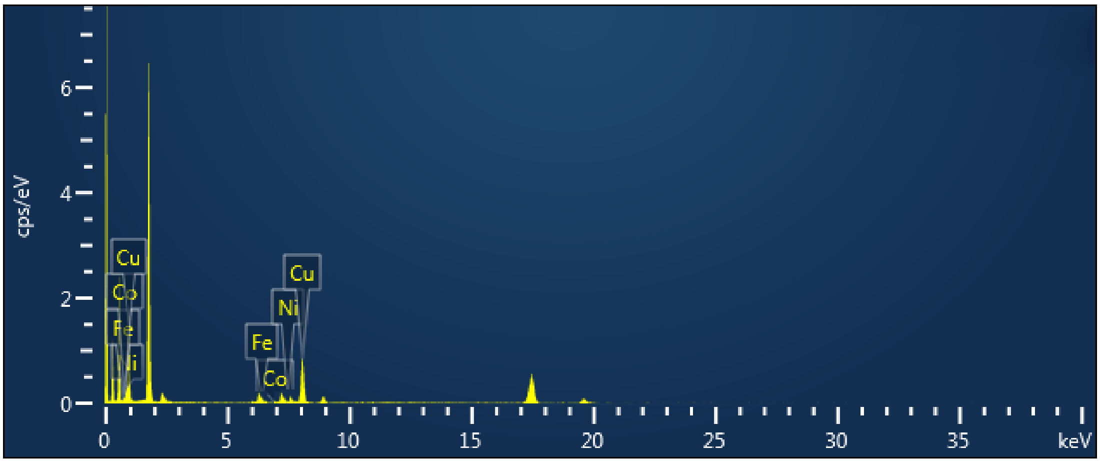

Element	Line Type	k Factor	k Factor type	Absorption Correction	Wt%	Wt% Sigma	Atomic %
**Fe**	K series	0.500		1.00	8.16	0.86	9.34
**Co**	K series	0.518		1.00	9.68	0.87	10.16
**Ni**	K series	0.509		1.00	9.77	1.01	10.27
**Cu**	K series	0.545		1.00	72.4	1.04	70.23
**Total:**					100.00		100.00

Figure S8. TEM-EDS analysis of the 8 h dealloyed secondary mesoporous structure.

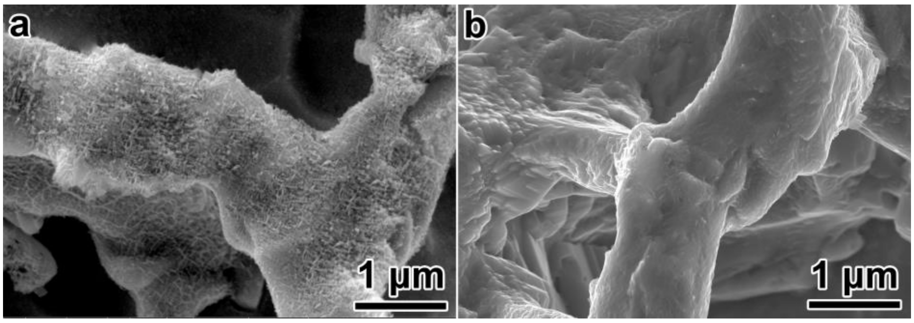

Figure S9. SEM images of the 8 h dealloyed FeCoNiCu HEAs fabricated by (a) casting and (b) rapid quenching (tube sealed and heated to 1200 ^{o}C) in water, respectively.

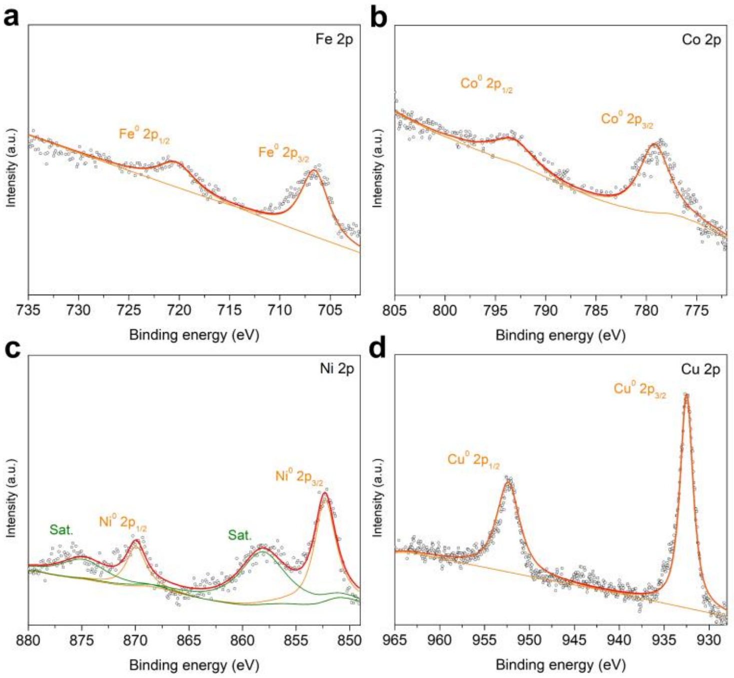

Figure S10. XPS spectra of the as-cast FeCoNiCu HEA: (**a**) Fe, (**b**) Co, (**c**) Ni, and (**d**) Cu.

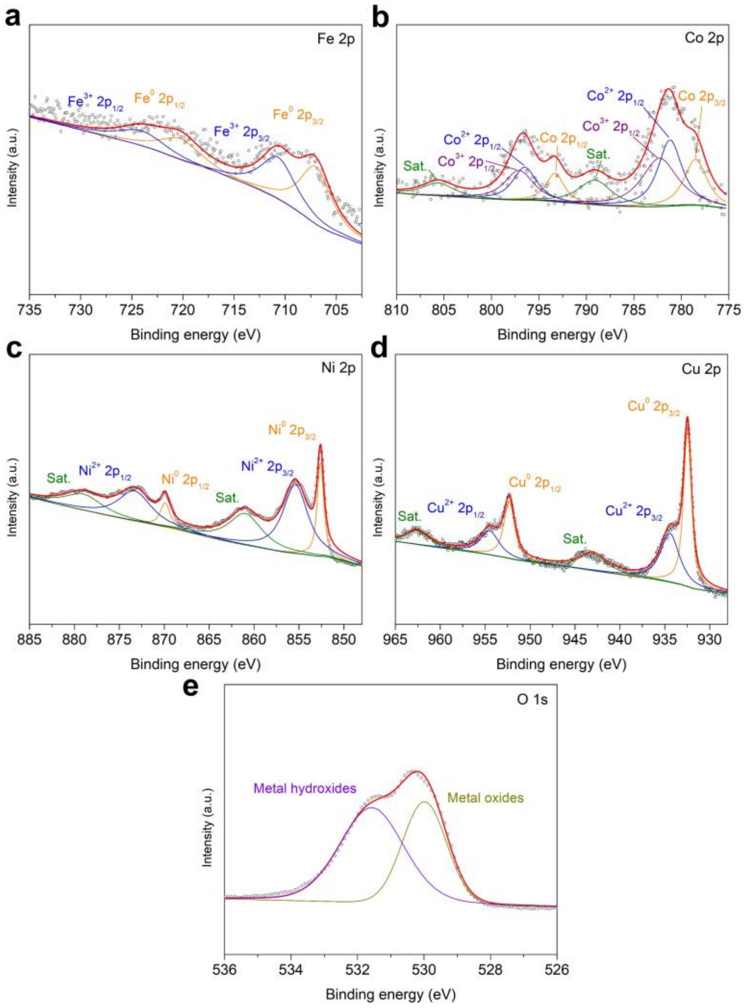

Figure S11. XPS spectra of the 8 h dealloyed FeCoNiCu HEA: (a) Fe, (b) Co, (c) Ni, (d) Cu, and (e) O.

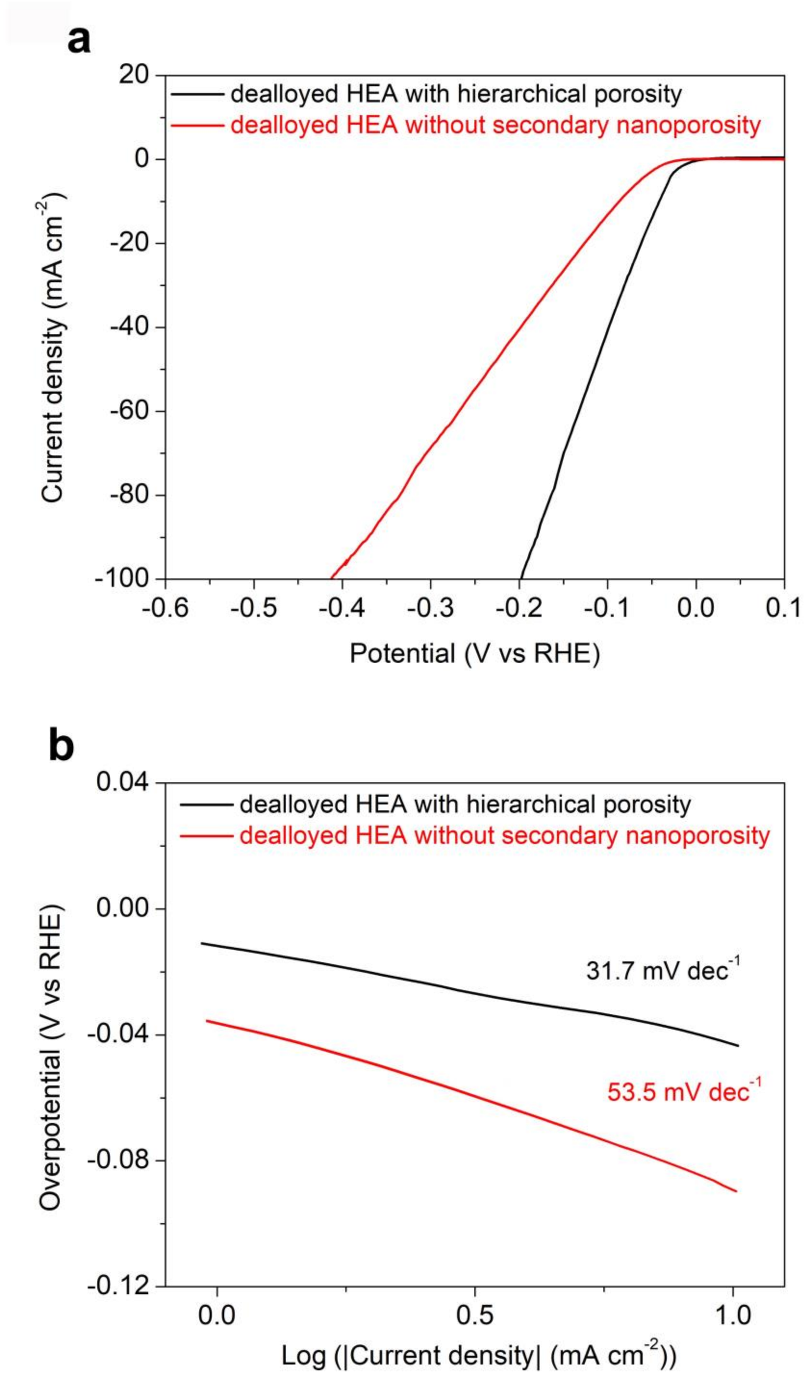

Figure S12. (a) Comparison of *iR*-corrected HER polarization curves of the 8 h dealloyed FeCoNiCu HEA catalysts with hierarchical porosity and without secondary nanoporosity in 1.0 $_{M}$ KOH aqueous electrolyte. (b) The corresponding Tafel slopes of the dealloyed HEAs.

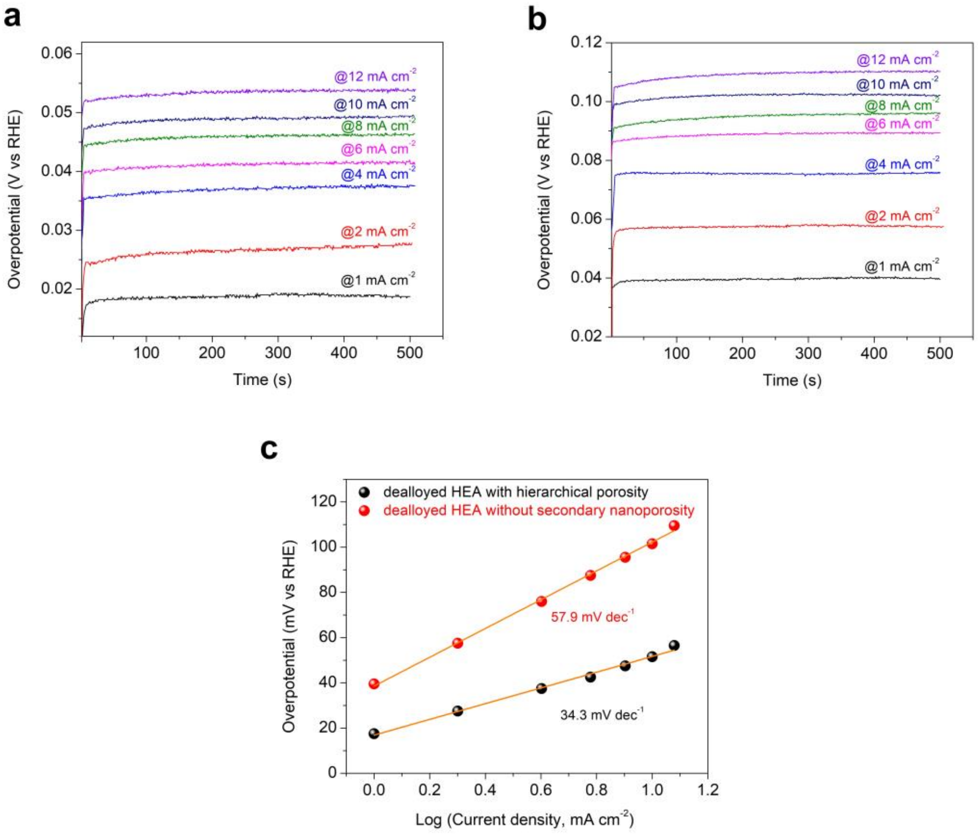

Figure S13. Steady-state galvanostatic responses of the 8 h dealloyed FeCoNiCu HEA catalysts (a) with and (b) without secondary nanoporosity under different current densities. The overpotentials were all corrected for *iR* losses. (c) Comparison of the true steady-state Tafel plot of the catalysts obtained from the galvanostatic response.

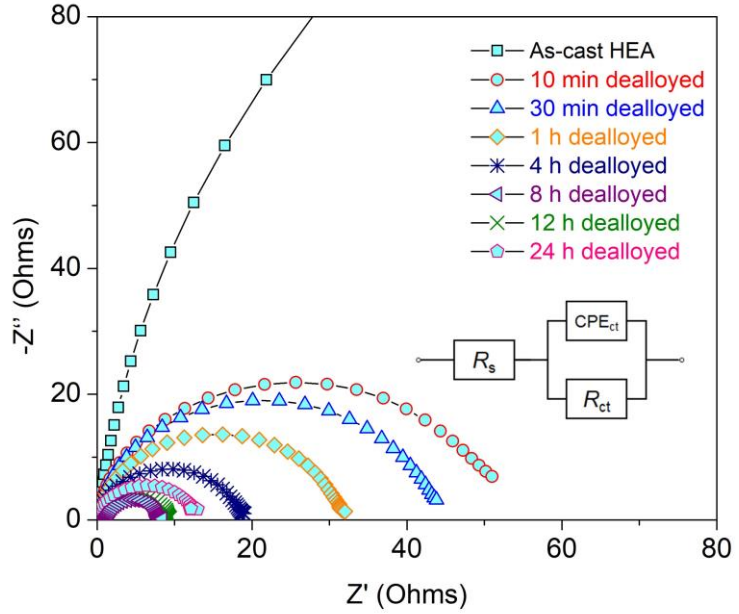

Figure S14. Nyquist plots of the dealloyed FeCoNiCu HEA electrodes with different dealloying times.

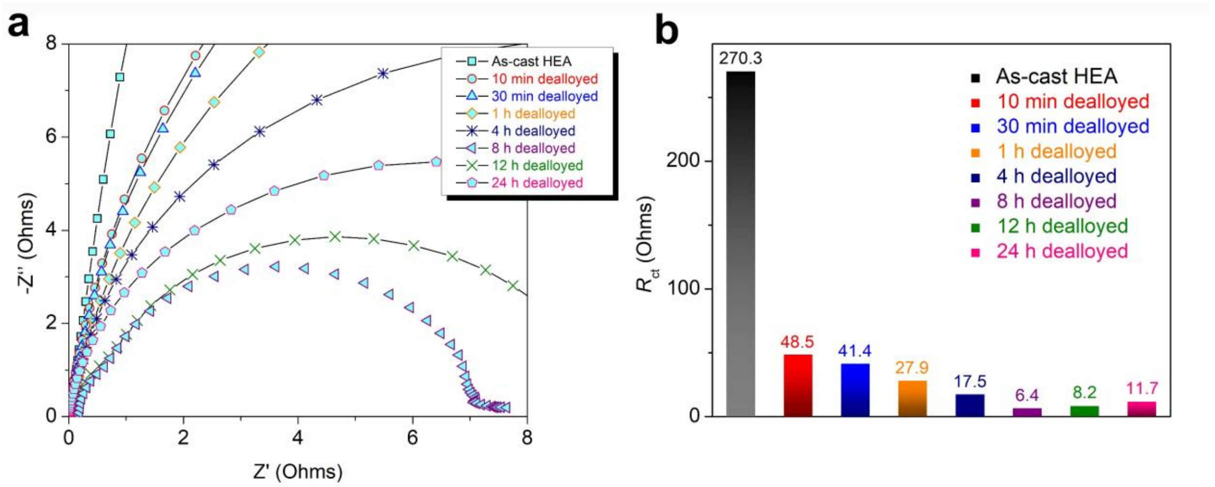

Figure S15. (a) High-frequency regions of the Nyquist plots of the dealloyed FeCoNiCu HEA electrodes with different dealloying times. (b) Comparison of *R*ct values of the dealloyed HEA samples.

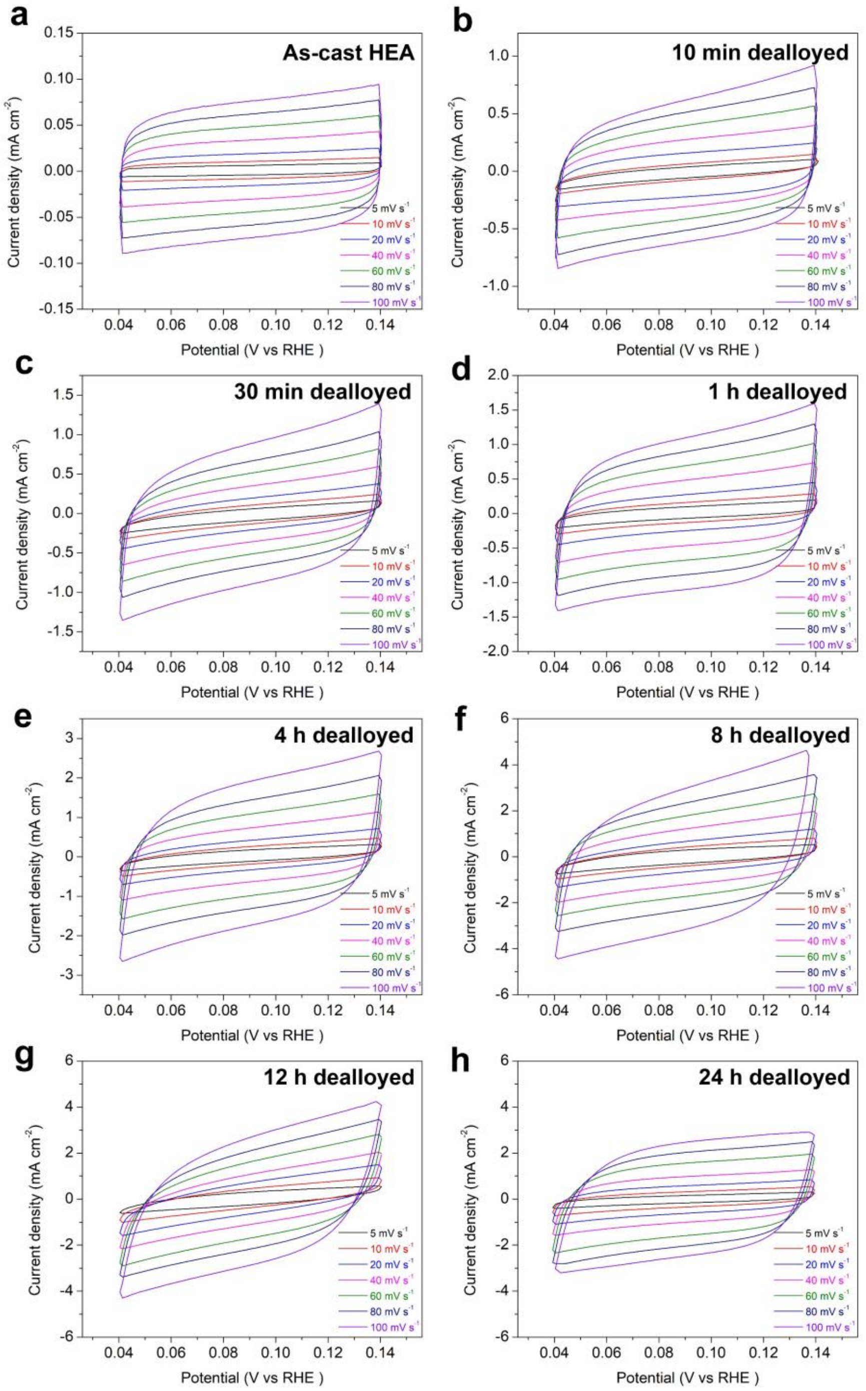

Figure S16. Cyclic voltammograms (CVs) of the dealloyed FeCoNiCu HEA with different etching durations in 0.1 M HNO_3 aqueous solution: (a) 0 min (i.e., the as-cast HEA), (b) 10 min, (c) 30 min, (d) 1 h, (e) 4 h, (f) 8 h, (g) 12 h and (h) 24 h. The CVs were performed at various scan rates (5, 10, 20, 40, 60, 80, and 100 mV s^{-1}) from 0.041 to 0.141 V vs RHE.

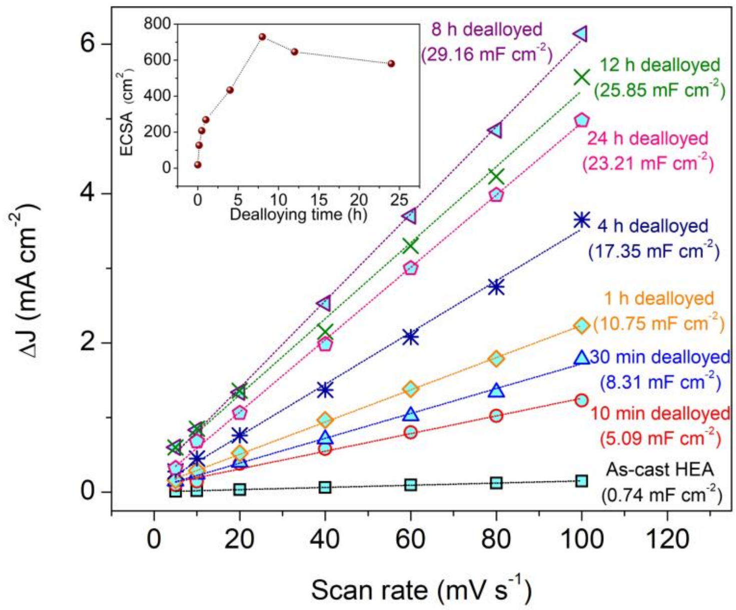

Figure S17. The plots of current densities against scan rates. △*j* is the difference between anodic and cathodic current density at a potential of 0.091 V vs RHE. The slope of the fitting line is twice of the electrochemical double-layer capacitance, which is proportional to the ECSA of the electrodes. The inset shows the dependence of ECSA on the dealloying time.

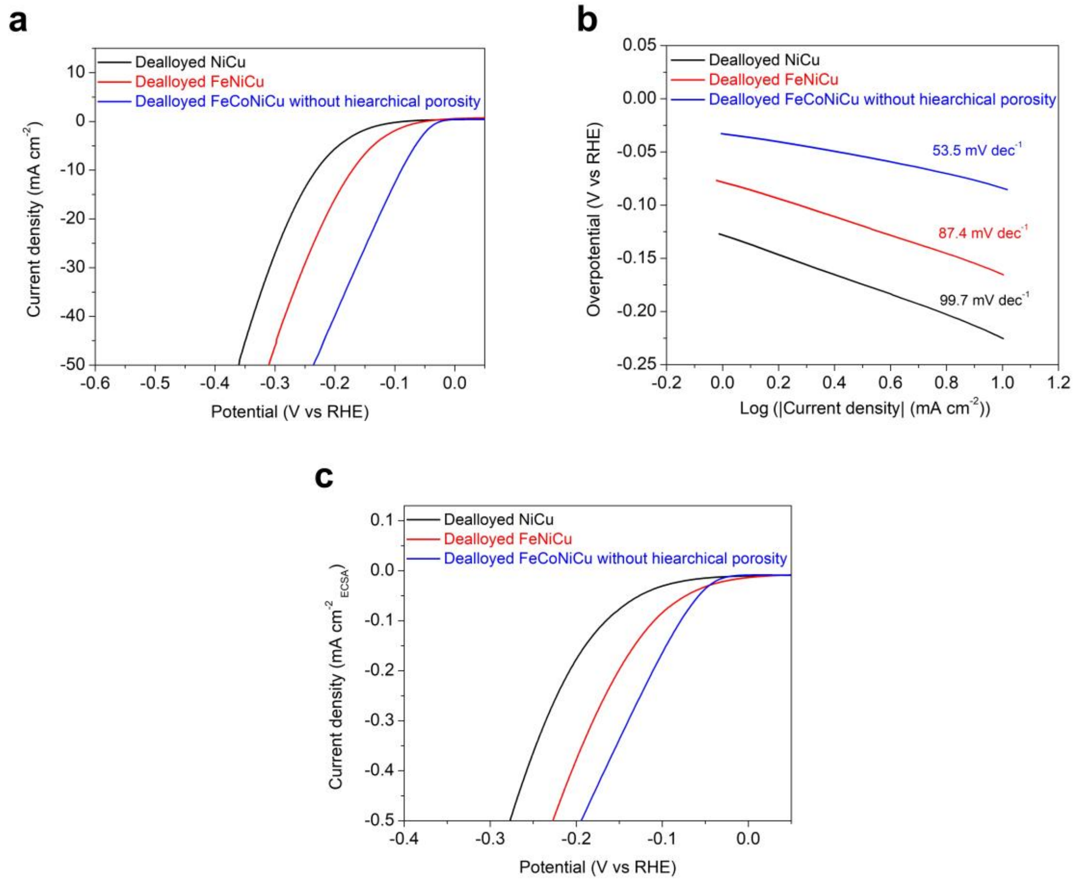

Figure S18. (a) Comparison of *iR*-corrected HER polarization curves of the dealloyed NiCu, FeNiCu and FeCoNiCu (without hierarchical porosity) alloy catalyst fabricated under the same conditions. (b) The corresponding Tafel slopes of these alloy catalysts. (c) The ECSA normalized HER activities of the three dealloyed catalysts, which further reflects the effect of the multicomponent alloying on the intrinsic activity.

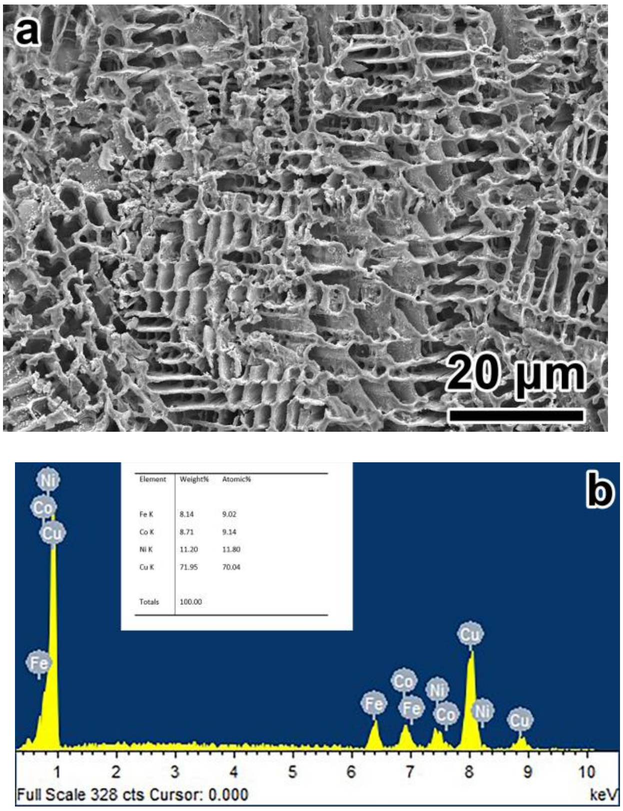

Figure S19. (a) Surface SEM image and (b) EDS analysis of the 8 h dealloyed FeCoNiCu HEA catalyst after the 120 h chrono-potentiometric stability test under a current density of 500 mA cm^{-2}.

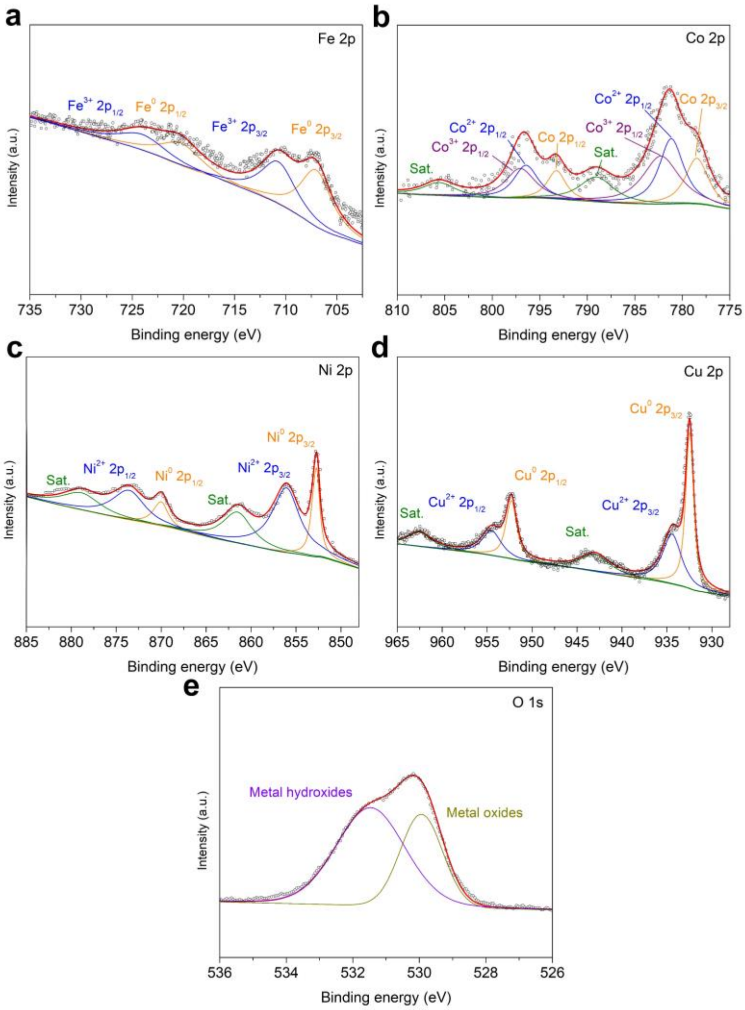

Figure S20. XPS spectra of the 8 h dealloyed FeCoNiCu HEA catalyst after the 120 h chrono-potentiometric stability: (a) Fe, (b) Co, (c) Ni, (d) Cu, and (e) O.

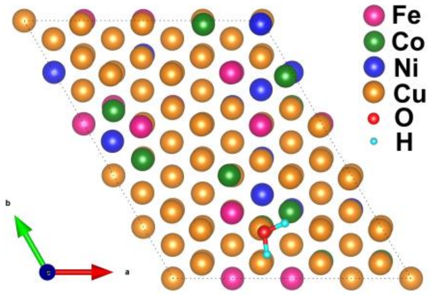

Figure S21. The atom configuration of H_2O molecule adsorption on the optimal Cu site of the $Fe_{10}Co_{10}Ni_{10}Cu_{70}$-(111) surface.

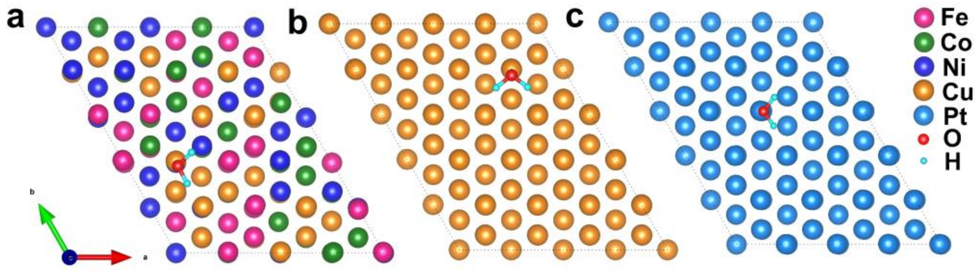

Figure S22. The atom configurations of H_2O molecule adsorption on the optimal active sites of the (a) $Fe_{25}Co_{25}Ni_{25}Cu_{25}$-(111), (b) Cu-(111), and (c) Pt-(111) surface.

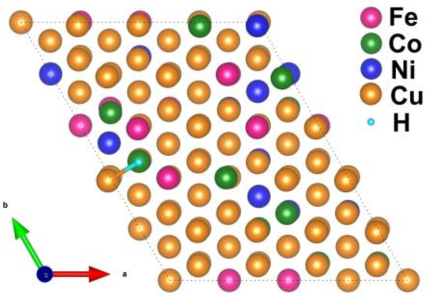

Figure S23. The atom configuration of H* adsorption on the optimal Cu site of the $Fe_{10}Co_{10}Ni_{10}Cu_{70}$-(111) surface.

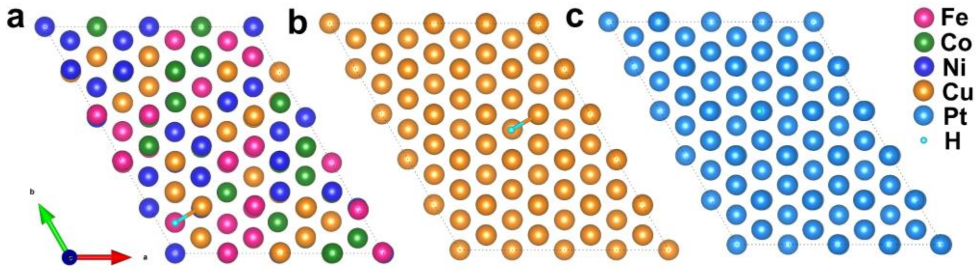

Figure S24. The atom configurations of H* adsorption on the optimal site of the (a) $Fe_{25}Co_{25}Ni_{25}Cu_{25}$-(111) face, (b) Cu-(111) face, and (c) Pt-(111) face.

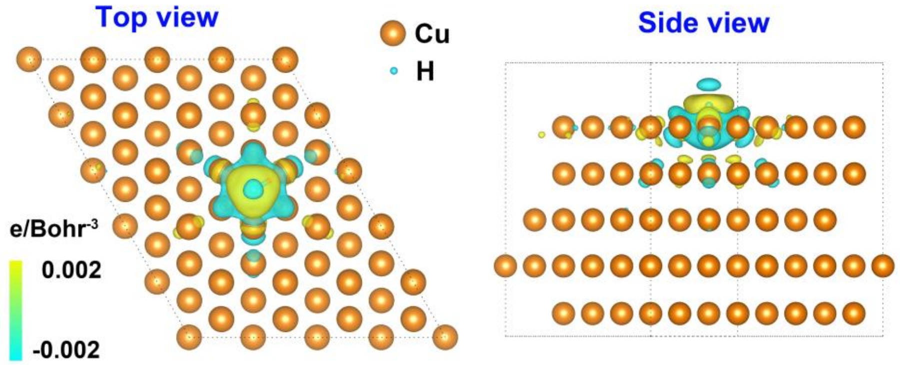

Figure S25. Computed differential charge densities for hydrogen adsorption on the Cu site of the pure Cu-(111) surface (Left: top view, right: side view). Yellow and blue bubbles represent the positive and negative charges with an iso-value of 0.002 e $Å^{-3}$, respectively.

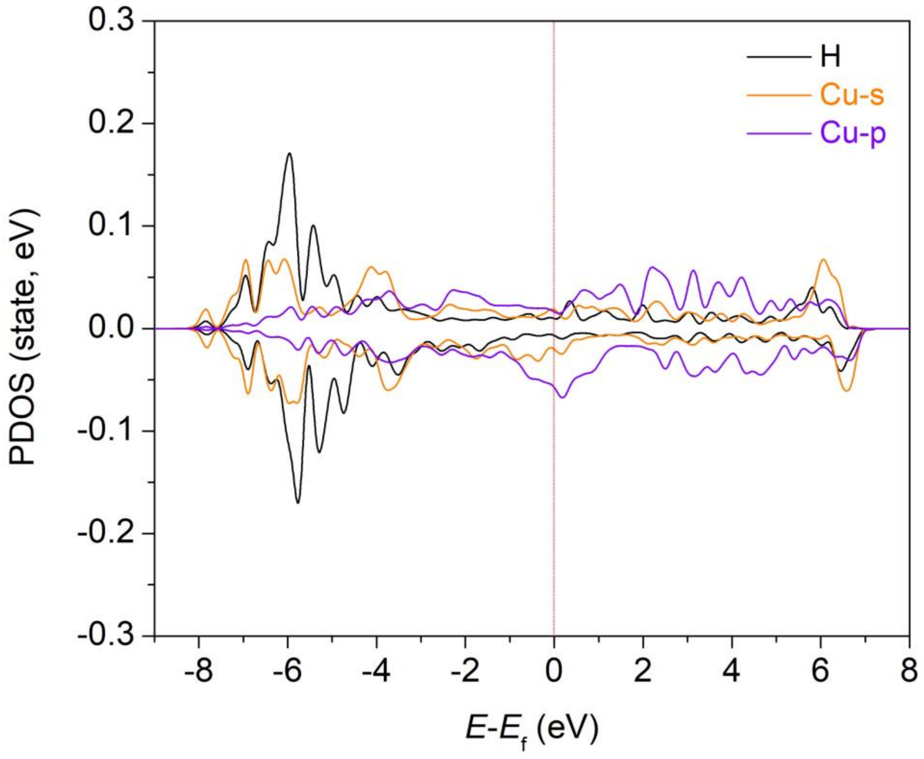

Figure S26. Partial density of states (PDOS) of the Cu-(111) surface after the H* adsorption on the optimal Cu site. The red dotted lines at the energy of zero indicate the Fermi level.

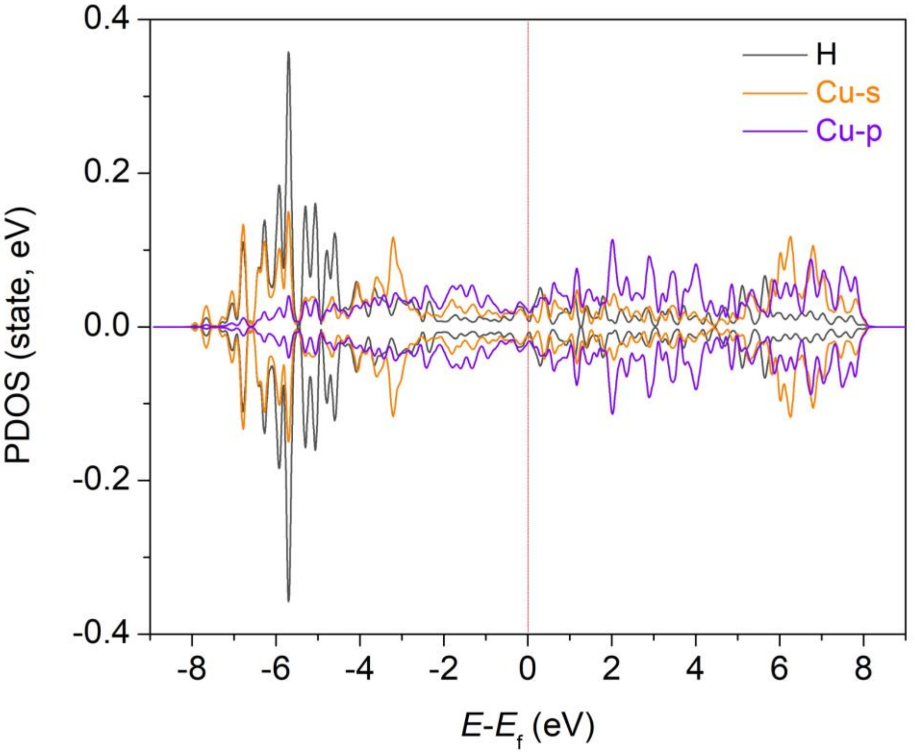

Figure S27. PDOS of the $Fe_{25}Co_{25}Ni_{25}Cu_{25}$-(111) surface after the H* adsorption on the optimal Cu site. The red dotted lines at the energy of zero indicate the Fermi level.

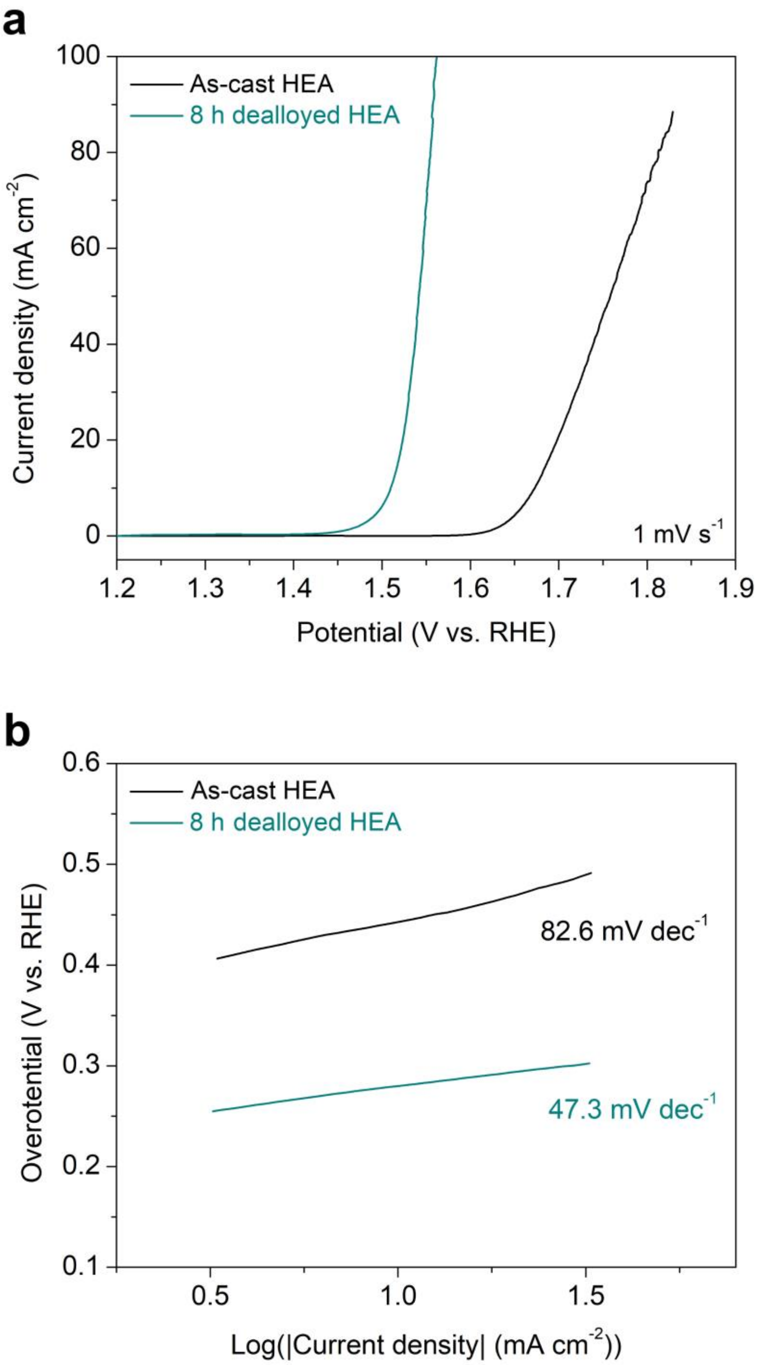

Figure S28. *iR*-corrected OER polarization curves (a) and Tafel slopes (b) of the as-cast and the 8 h dealloyed FeCoNiCu HEA in 1.0 $_{\mathrm{M}}$ KOH electrolyte.

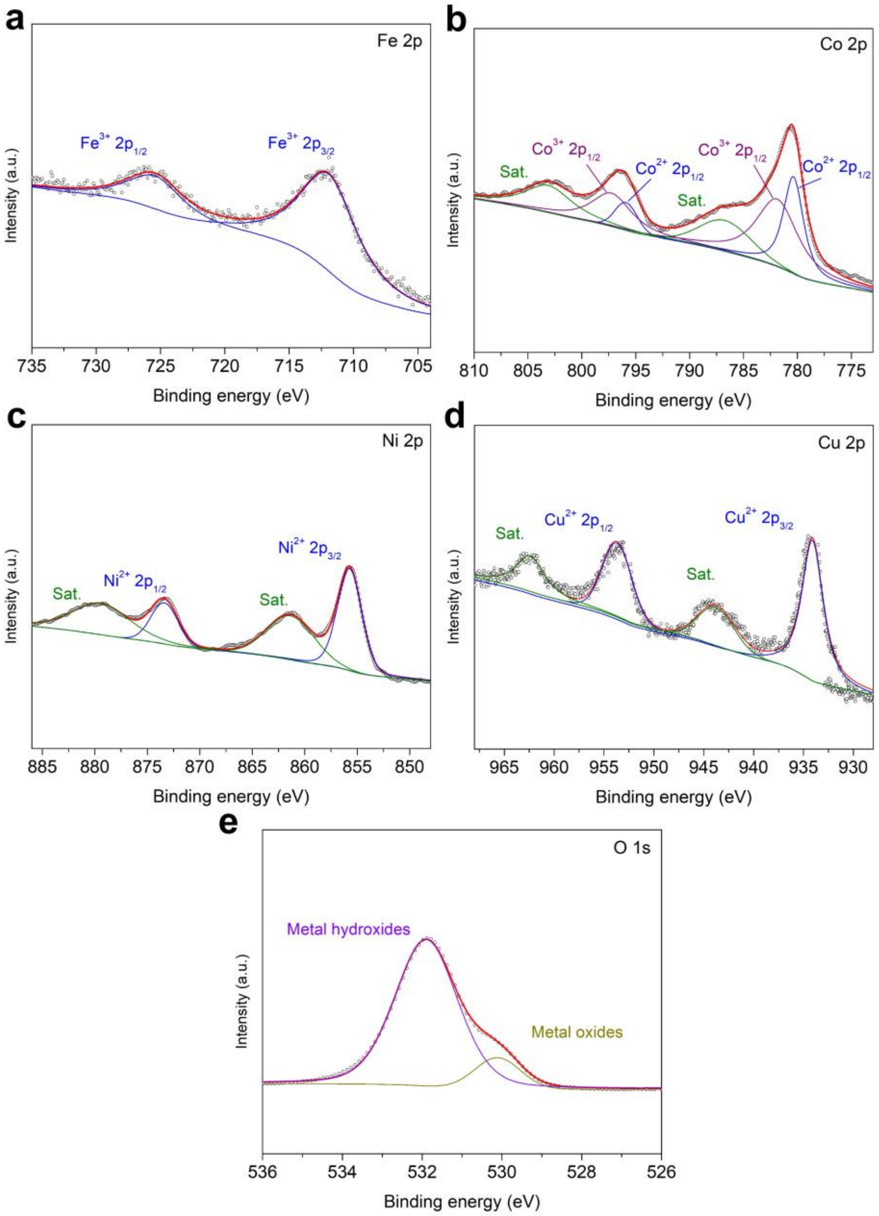

Figure S29. XPS spectra of the 8 h dealloyed FeCoNiCu HEA on the OER electrode after the 240 h-chronopotentiometric stability test: (a) Fe, (b) Co, (c) Ni, (d) Cu, and (e) O.

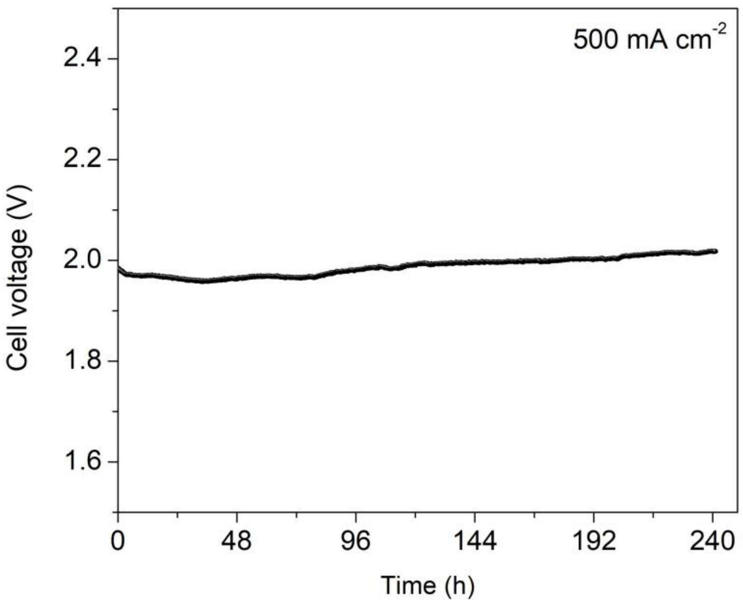

Figure S30. Chronopotentiometry durability curves of the dealloyed HEA||dealloyed HEA measured in 1 M KOH at the current density of 500 mA cm^{-2}.

Table S1. ICP analyses of the FeCoNiCu HEA samples before and after 8 h dealloying in 0.1 M HNO_3 aqueous solution.

Solution	***Co (238.892 nm)***	***Cu (327.395 nm)***	***Fe (238.204 nm)***	***Ni (231.604 nm)***
Standard 1	3.00 (ppm)	3.00 (ppm)	3.00 (ppm)	3.00 (ppm)
Standard 2	6.00 (ppm)	6.00 (ppm)	6.00 (ppm)	6.00 (ppm)
Standard 3	9.00 (ppm)	9.00 (ppm)	9.00 (ppm)	9.00 (ppm)
HEA-Before dealloying	247603.17 (ppm)	273658.65 (ppm)	233519.30 (ppm)	24.4901.26 (ppm)
Calculated wt.	**24.77%**	**27.36%**	**23.37%**	**24.49%**
Calculated at.	**24.92%**	**25.53%**	**24.81%**	**24.74%**
HEA-After dealloying	95357.26 (ppm)	716662.11 (ppm)	86862.74 (ppm)	101425.71 (ppm)
Calculated wt.	**9.54%**	**71.67%**	**8.69%**	**10.1%**
Calculated at.	**10.01%**	**69.73%**	**9.62%**	**10.64%**

Table S2. Comparison of the HER performances for the dealloyed HEA material with previously reported electrocatalysts in alkaline electrolytes.

Electrocatalysts	Substrate	Electrolyte	Overpotential (mV) at 10 mA cm^{-2}	Tafel slope (mV dec^{-1})	Ref.
**8 h dealloyed FeCoNiCu HEA**	**Free-standing**	**1.0 M KOH**	**42.2**	**31.7**	**This work**
**Dealloyed FeNiCoAlTi HEA**	Free-standing	1.0 M KOH	88.2	40.1	[1]
**NiCuPtPdAu nanoporous HEA**	Glassy carbon	0.1 M KOH	~38	47	[2]
**PtAuPdRhRu HEA nanoparticles**	Disk electrode	1.0 M KOH	~90	62	[3]
**Annealed NiFeMoCoCr HEA**	Free-standing	1.0 M KOH	172	66	[4]
**Nanoporous NiMnFeMo**	Free-standing	1.0 M KOH	67 (100 mA cm^{-2})	32	[5]
**FeCoCrMnNiP HEMP**	Glassy carbon	1.0 M KOH	136	85.5	[6]
**IrPdPtRhRu HEA nanoparticles**	Glassy carbon	1.0 M KOH	17	/	[7]
**PdFeCoNiCu HEA nanoparticles**	Glassy carbon	1.0 M KOH	18	39	[8]
**Nanoporous CuAlNiMoFe HEA**	Free-standing	1.0 M KOH	9.7	60	[9]
**Nanoporous Ni-Pt/ metallic glass (MG)**	Free-standing	1.0 M KOH	37	30	[10]
**Nanoporous Ni/MG**	Free-standing	1.0 M KOH	78	42.4	[11]
**$Ni_{40}Fe_{40}P_{20}$ MG**	Free-standing	1.0 M KOH	270	89	[12]
**Ultrasonic vibration treated $Fe_{78}Si_9B_{13}$ MG**	Free-standing	1.0 M KOH	173	169	[13]
**Amorphous NiCoFeP**	Ni foam	1.0 M KOH	56	83.2	[14]
**Amorphous RuFeP/ carbon nanofiber**	Glassy carbon	1.0 M KOH	16	40.31	[15]
**Cr- doped FeNi-P/NCN**	Glassy carbon	1.0 M KOH	190	68.51	[16]
**$FeNi_3$-$FeNi_3N$**	Glassy carbon	1.0 M KOH	51	83	[17]
**FeNi@NCF**	Glassy carbon	1.0 M KOH	219	109.9	[18]
**$FeNi_3N$/NG**	Glassy carbon	1.0 M KOH	98 (20 mA cm^{-2})	83.1	[19]

**NiFe LDH-NS@DG**	Glassy carbon	1.0 M KOH	115 (20 mA cm^{-2})	110	[20]
**NiFe sponge**	Glassy carbon	1.0 M KOH	190	82	[21]
**NiFe LDH- POM/NF**	Ni foam	0.1 M KOH	156	86	[22]
**Ni_3N/NF**	Ni foam	1.0 M KOH	44	46	[23]
**$FeCoPO_4$**	Ni foam	1.0 M KOH	77	80.7	[24]
**Ni-Co-P HNBs**	Ni foam	1.0 M KOH	107	46	[25]
**Fe-Ni_3S_2**	$FeNi_3$ foam	1.0 M KOH	105	69	[26]
**Nanoporous CoP_3**	Ti mesh	0.1 M KOH	76	45	[27]
**Nanoporous Co_2P**	Free-standing	1.0 M KOH	80	44	[28]
**Nanoporous CoSe with Single Pt atoms**	Glassy carbon	1.0 M KOH	58	39	[29]
**Co/CoO hybrid**	Carbon cloth	1.0 M KOH	158	68.1	[30]
**Co-Cu-WS_x nanosphere**	Glassy carbon	1.0 M KOH	82.5	53.8	[31]
**Co_3Mo/nanoporous Cu**	Free-standing	1.0 M KOH	12	40	[32]
**CuO-Co_3O_4 nanocomposite**	Glassy carbon	1.0 M KOH	288	65	[33]
**NC@$CuCoN_x$/CF**	Glassy carbon	1.0 M KOH	105	76	[34]
**AlCuNi/nanoporous Cu**	Free-standing	0.1 M KOH	139	110	[35]
**RuP Nanoparticles**	Glassy carbon	1.0 M KOH	18	34	[36]
**Nanoporous PdAg**	Free-standing	1.0 M KOH	16	56	[37]
**AuRu nanowires**	Glassy carbon	1.0 M KOH	25	15.6	[38]
**Ru@graphene nanoplatelets**	Glassy carbon	1.0 M KOH	22	28	[39]
**PtRu NCS/BP**	Disk electrode	1.0 M KOH	22	19	[40]
**Ru-Ru_2P/PC**	Glassy carbon	1.0 M KOH	50	35.1	[41]
**RhPdH nanosheets**	Glassy carbon	1.0 M KOH	40	35.7	[42]
**IrMo nanocatalysts**	Glassy carbon	0.1 M KOH	38	60	[43]
**RhO_2 clusters**	Glassy carbon	1.0 M KOH	33	39	[44]
**Ir@CON**	Disk electrode	1.0 M KOH	13.5	29	[45]

Table S3. Calculation of Faradaic efficiency

	Time (min)	Detected H_2 (umol)	Faradaic efficiency (H_2, %)
**Current density: 10 mA cm^{-2}**	20	62.041	99.6
	40	123.265	99.2
	60	184.490	98.9
	80	245.714	98.8
	100	308.163	99.1
	120	370.204	99.2

The Faradaic efficiency of the porous HEA-based catalyst towards HER in 1.0 $_{M}$ KOH electrolyte was measured in a closed electrochemical cell and the collected H_2 gas products were analyzed by gas chromatography (Shimadzu, GC-2014). Each collection was repeated at least three times, and the average value is presented. Then, the value of the Faradaic efficiency was calculated based on the following formula:

$$Faradaic\ efficiency\ (\mathrm{H_2},\ \%) = \frac{V_{\mathrm{H_2}} \times 2 \times F}{V_{\mathrm{m}} \times j \times t} \times 100\%$$

V_{H2} is the detected volume of hydrogen, F is the Faraday constant (96485.33289 C mol^{-1}), V_m is the molar volume of the gas (24.5 L mol^{-1}, 25 ^{o}C), j is the current density (10 mA cm^{-2}) and t is the time period of the electrolysis.

Table S4. Calculated water adsorption energies on different active sites of the catalyst surface.

Catalyst surface	Adsorption sites	$E_{surface}$ (eV)	E_{H_2O} (eV)	$E_{H_2O\_ads\_surface}$ (eV)	E_{ads} (eV)
**$Fe_{70}Co_{10}Ni_{10}Cu_{10}$ -(111)**	site 1#	-471.111	-14.224	-485.764	-0.428
	site 2#	-471.111	-14.224	-485.676	-0.340
	site 3#	-471.111	-14.224	-485.747	-0.411
	site 4#	-471.111	-14.224	-485.798	-0.463
	site 5#	-471.111	-14.224	-485.791	-0.456
**$Fe_{25}Co_{25}Ni_{25}Cu_{25}$ -(111)**	site 1#	-606.183	-14.224	-620.778	-0.370
	site 2#	-606.183	-14.224	-620.825	-0.417
	site 3#	-606.183	-14.224	-619.912	0.496
	site 4#	-606.183	-14.224	-620.871	-0.463
	site 5#	-606.183	-14.224	-620.733	-0.326
**Cu-(111)**	site 1#	-392.346	-14.224	-406.999	-0.429
	site 2#	-392.346	-14.224	-407.001	-0.430
	site 3#	-392.346	-14.224	-407.004	-0.434
	site 4#	-392.346	-14.224	-407.003	-0.433
**Pt-(111)**	site 1#	-642.671	-14.224	-657.418	-0.523
	site 2#	-642.671	-14.224	-657.423	-0.528
	site 3#	-642.671	-14.224	-657.419	-0.524
	site 4#	-642.671	-14.224	-657.416	-0.521

$E_{surface}$, E_{H_2O}, $E_{H_2O\_ads\_surface}$, and E_{ads} represent the energy of the clean surface, the energy of H_2O, the energy of H_2O adsorbed on the surface, and the H_2O adsorption energy, respectively.

Table S5. Calculated hydrogen adsorption Gibbs free energies on the optimal active site of the catalyst surface.

Catalyst surface	Optimal adsorption sites	ΔE_{H^*} (eV)	ΔG_{H^*} (eV)
**$Fe_{70}Co_{10}Ni_{10}Cu_{10}$ -(111)**	*Cu site (hcp)*	-0.645	0.377
**$Fe_{25}Co_{25}Ni_{25}Cu_{25}$ -(111)**	*Cu site (hcp)*	-0.621	0.398
**Cu-(111)**	*fcc*	-0.545	0.716
**Pt-(111)**	*top*	-0.303	0.484

ΔE_{H^*} and ΔG_{H^*} represent the DFT electronic energy difference of each step and the hydrogen adsorption Gibbs free energy which is corrected by the PH value ($\Delta G=0.0591 \times PH$), respectively.

References

[1] Z. Jia, T. Yang, L. Sun, Y. Zhao, W. Li, J. Luan, F. Lyu, L. C. Zhang, J. J. Kruzic, J. J. Kai, *Adv. Mater.* **2020,** *32*, 2000385.

[2] H.-J. Qiu, G. Fang, Y. Wen, P. Liu, G. Xie, X. Liu, S. Sun, *J. Mater. Chem. A* **2019,** *7*, 6499.

[3] M. Liu, Z. Zhang, F. Okejiri, S. Yang, S. Zhou, S. Dai, *Adv. Mater. Interfaces* **2019,** *6*, 1900015.

[4] G. Zhang, K. Ming, J. Kang, Q. Huang, Z. Zhang, X. Zheng, X. Bi, *Electrochim. Acta* **2018,** *279*, 19.

[5] H. Liu, C. Xi, J. Xin, G. Zhang, S. Zhang, Z. Zhang, Q. Huang, J. Li, H. Liu, J. Kang, *Chem. Eng. J.* **2021,** *404*, 126530.

[6] X. Zhao, Z. Xue, W. Chen, Y. Wang, T. Mu, *ChemSusChem* **2020,** *13*, 2038.

[7] D. Wu, K. Kusada, T. Yamamoto, T. Toriyama, S. Matsumura, I. Gueye, O. Seo, J. Kim, S. Hiroi, O. Sakata, *Chem. Sci.* **2020,** *11*, 12731.

[8] D. Zhang, Y. Shi, H. Zhao, W. Qi, X. Chen, T. Zhan, S. Li, B. Yang, M. Sun, J. Lai, B. Huang, L. Wang, *J. Mater. Chem. A* **2021,** *9*, 889.

[9] R.-Q. Yao, Y.-T. Zhou, H. Shi, W.-B. Wan, Q.-H. Zhang, L. Gu, Y.-F. Zhu, Z. Wen, X.-Y. Lang, Q. Jiang, Advanced Functional Materials, *Adv. Funct. Mater.* **2021**, *31*. 2009613.

[10] R. Li, X. Liu, R. Wu, J. Wang, Z. Li, K. C. Chan, H. Wang, Y. Wu, Z. Lu, *Adv. Mater.* **2019,** (49), 1904989.

[11] J. Wang, L. You, Z. Li, X. Liu, R. Li, Q. Du, X. Wang, H. Wang, Y. Wu, S. Jiang, Z. Lu, *J. Mater. Sc. Technol.* **2021,** *73*, 145.

[12] Y. Tan, F. Zhu, H. Wang, Y. Tian, A. Hirata, T. Fujita, M. Chen, *Adv. Mater. Interfaces* **2017,** *4*, 1601086.

[13] K. Wu, Y. Meng, X. Li, J. Ma, P. Zhang, W. Li, L. Huo, H.-J. Lin, *Intermetallics* **2020,** *125*, 106820.

[14] K. Wang, Y. Si, Z. Lv, T. Yu, X. Liu, G. Wang, G. Xie, L. Jiang, *Int. J. Hydrogen Energ.* **2020,** *45*, 2504.

[15] B. Yang, J. Xu, D. Bin, J. Wang, J. Zhao, Y. Liu, B. Li, X. Fang, Y. Liu, L. Qiao, L. Liu, B. Liu, *Appl. Catal. B: Environ.* **2021,** *283*, 119583.

[16] Y. Wu, X. Tao, Y. Qing, H. Xu, F. Yang, S. Luo, C. Tian, M. Liu, X. Lu, *Adv. Mater.* **2019,** *31*, 1900178.

[17] S. Liang, M. Jing, T. Thomas, J. Liu, H. Guo, J. P. Attfield, A. Saad, H. Shen, M. Yang, *Sustain. Energ. Fuels* **2020,** *4*, 6245.

[18] Z. Zhang, L. Cong, Z. Yu, L. Qu, W. Huang, *Mater. Today Energy* **2020,** *16*, 100387.

[19] L. Liu, F. Yan, K. Li, C. Zhu, Y. Xie, X. Zhang, Y. Chen, *J. Mater. Chem. A* **2019,** *7*, 1083.

[20] Y. Jia, L. Zhang, G. Gao, H. Chen, B. Wang, J. Zhou, M. T. Soo, M. Hong, X. Yan, G. Qian, *Adv. Mater.* **2017,** *29*, 1700017.

[21] S. Thoufeeq, P. K. Rastogi, S. Thomas, A. Shravani, T. N. Narayanan, M. R. Anantharaman, *ChemistrySelect* **2020,** *5*, 1385.

[22] C. Li, Z. Zhang, R. Liu, *Small* **2020,** *16*, 2003777.

[23] G. Li, X. Wu, H. Guo, Y. Guo, H. Chen, Y. Wu, J. Zheng, X. Li, *ACS Appl. Mater. Inter.* **2020,** *12*, 5951.

[24] C. Li, X. Mei, F. L.-Y. Lam, X. Hu, *ACS Appl. Energy Mater.* **2018,** *1*, 6764.

[25] E. Hu, Y. Feng, J. Nai, D. Zhao, Y. Hu, X. W. D. Lou, E *Energy & Environ. Sci.* **2018,** *11*, 872.

[26] W. Zhang, Q. Jia, H. Liang, L. Cui, D. Wei, J. Liu, *Chem. Eng. J.* **2020,** *396*, 125315.

[27] Y. Ji, L. Yang, X. Ren, G. Cui, X. Xiong, X. Sun, *ACS Sustain. Chem. Eng.* **2018,** *6*, 11186.

[28] Y. Tan, H. Wang, P. Liu, C. Cheng, F. Zhu, A. Hirata, M. Chen, *Adv. Mater.* **2016,** *28*, 2951.

[29] K. Jiang, B. Liu, M. Luo, S. Ning, M. Peng, Y. Zhao, Y.-R. Lu, T.-S. Chan, F. M. F. de Groot, Y. Tan, *Nature Commun.* **2019,** *10*, 1743.

[30] J. Cao, X. Chen, H. Li, J. Pu, L. Liu, L. Ma, K. Zhou, Z. Zhang, Q. Wei, F. Luo, *Sustain. Energ. Fuels* **2020,** *4*, 1924.

[31] X. Qian, H. Liu, J. Yang, H. Wang, J. Huang, C. Xu, *J. Mater. Chem. A* **2019,** *7*, 6337.

[32]H. Shi, Y.-T. Zhou, R.-Q. Yao, W.-B. Wan, X. Ge, W. Zhang, Z. Wen, X.-Y. Lang, W.-T. Zheng, Q. Jiang, *Nature Commun.* **2020,** *11*, 2940.

[33] A. Tahira, Z. H. Ibupoto, M. Willander, O. Nur, *Int. J. Hydrogen Energ.* **2019,** *44*, 26148.

[34] J. Zheng, X. Chen, X. Zhong, S. Li, T. Liu, G. Zhuang, X. Li, S. Deng, D. Mei, J.-G. Wang, *Adv. Funct. Mater.* **2017,** *27*, 1704169.

[35] J. S. Sun, Z. Wen, L. P. Han, Z. W. Chen, X. Y. Lang, Q. Jiang, *Adv. Funct. Mater.* **2018,** *28*, 1706127.

[36] J. Yu, Y. Guo, S. She, S. Miao, M. Ni, W. Zhou, M. Liu, Z. Shao, *Adv. Mater.* **2018,** *30*, 1800047.

[37] R.-Q. Yao, Y.-T. Zhou, H. Shi, Q.-H. Zhang, L. Gu, Z. Wen, X.-Y. Lang, Q. Jiang, *ACS Energy*

Lett. **2019,** *4*, 1379.

[38] Q. Lu, A.-L. Wang, Y. Gong, W. Hao, H. Cheng, J. Chen, B. Li, N. Yang, W. Niu, J. Wang, *Nat. Chem.* **2018,** *10*, 456.

[39] F. Li, G.-F. Han, H.-J. Noh, I. Ahmad, I.-Y. Jeon, J.-B. Baek, *Adv. Mater.* **2018,** *30*, 1803676.

[40] Y. Li, W. Pei, J. He, K. Liu, W. Qi, X. Gao, S. Zhou, H. Xie, K. Yin, Y. Gao, J. He, J. Zhao, J. Hu, T.-S. Chan, Z. Li, G. Zhang, M. Liu, *ACS Catal.* **2019,** *9*, 10870.

[41] Z. Liu, Z. Li, J. Li, J. Xiong, S. Zhou, J. Liang, W. Cai, C. Wang, Z. Yang, H. Cheng, *J. Mater. Chem. A* **2019,** *7* 5621.

[42] J. Fan, J. Wu, X. Cui, L. Gu, Q. Zhang, F. Meng, B.-H. Lei, D. J. Singh, W. Zheng, *J. Am. Chem. Soc.* **2020,** *142*, 3645.

[43] L. Fu, Y. Li, N. Yao, F. Yang, G. Cheng, W. Luo, *ACS Catal.* **2020,** *10*, 7322.

[44] Z. Li, Y. Feng, Y.-L. Liang, C.-Q. Cheng, C.-K. Dong, H. Liu, X.-W. Du, *Adv. Mater.* **2020,** *32*, 1908521.

[45] J. Mahmood, M. A. R. Anjum, S.-H. Shin, I. Ahmad, H.-J. Noh, S.-J. Kim, H. Y. Jeong, J. S. Lee, J.-B. Baek, *Adv. Mater.* **2018,** *30*, 1805606.
